# Cyclovirobuxine D ameliorates cardiomyocyte senescence in diabetic cardiomyopathy mice by enhancing mitochondrial function via sirtuin 3–ATP5O signal axis

**DOI:** 10.1186/s13020-025-01254-3

**Published:** 2025-11-13

**Authors:** Jiang-fei An, Hang Su, Xue-ting Wang, Guang-qiong Zhang, Chao-da Xiao, BarBu Wollenberga, Yong-xin Chen, Hong Yang, Hong Luo, Long Yang, Ling-yun Fu, Yi-ni Xu, Ling Tao, Xiang-chun Shen

**Affiliations:** 1 The State Key Laboratory of Functions and Applications of Medicinal Plants, Guizhou Medical University, No.6 Ankang Avenue, Guian New District, Guiyang, 561113 Guizhou China; 2 Department of Pharmacy, Shi Zhen College of Guizhou University of Traditional Chinese Medicine, Guiyang, 550200 Guizhou China; 3 The High Efficacy Application of Natural Medicinal Resources Engineering Center of Guizhou Province, School of Pharmaceutical Sciences, Guizhou Medical University, No.6 Ankang Avenue, Guian New District, Guiyang, 561113 Guizhou China; 4 The Key Laboratory of Optimal Utilization of Natural Medicine Resources, School of Pharmaceutical Sciences, Guizhou Medical University, No.6 Ankang Avenue, Guian New District, Guiyang, 561113 Guizhou China; 5 Department of Pharmacy, Guiyang Maternal and Child Health Care Hospital, Guiyang, 550003 Guizhou China

**Keywords:** Cyclovirobuxine D, Primary mouse cardiomyocytes, Diabetic cardiomyopathy, Mitochondrial function, Sirtuin 3–ATP5O

## Abstract

**Background:**

Diabetic cardiomyopathy (DCM) is a cardiovascular complication, with cardiomyocyte senescence being a key pathological process. Cyclovirobuxine D (CVB-D), the active compound in *Buxus sinica* (Rehd. et Wils.) var. *parvifolia* M. Cheng. CVB-D has potentially promising diabetes-related cardiomyocyte senescence-mitigating effects. Nevertheless, the impact of CVB-D on inhibiting cardiomyocyte senescence has not been widely investigated and molecular mechanisms remain ambiguous.

**Methods:**

A diabetic mouse model was established via a high-fat diet (HFD) combined with streptozotocin (STZ). Sirtuin 3 (SIRT3) knockout, SIRT3 overexpression, and ATP5O knockout mouse models were constructed through 4-week intravenous injections of AAV9-U6-SIRT3, AAV9-CMV-SIRT3, AAV9-U6-ATP5O, and their negative controls (AAV9-CMV-NC and AAV9-U6-NC). A primary mice cardiomyocytes (NMVMs) senescence model was developed using high palmitic acid and high glucose (PA/HG)*.* Western blotting, reverse transcription-quantitative PCR (qRT-PCR), immunofluorescence, β-galactosidase staining and flow cytometry were performed to determine the protective role of CVB-D against cardiomyocyte senescence. The underlying mechanisms of CVB-D were investigated via molecular docking, coimmunoprecipitation (Co-IP), microscale thermophoresis (MST), surface plasmon resonance (SPR) binding assay, isothermal titration calorimetry (ITC) and LC–MS/MS analysis.

**Results:**

CVB-D treatment improves mitochondrial dysfunction in DCM and thus alleviates the aging of cardiomyocytes in vitro and in vivo. And then, the results revealed that CVB-D can upregulate the acetylation level of ATP5O by upregulating the expression of SIRT3 to alleviate cardiomyocyte senescence. The results of Co-IP, MST, SPR, and ITC, among other experiments revealed that CVB-D plays a functional role through the SIRT3**–**ATP5O axis. Potential ATP5O acetylation sites by the LC–MS/MS analysis, we found that SIRT3 deacetylates the K162 site of ATP5O in primary mouse cardiomyocytes. Furthermore, transfection with a deacetylated or acetylated mimic plasmid containing ATP5O decreased or promoted mitochondrial damage, respectively. SIRT3 overexpression ameliorated DCM, whereas ATP5O knockout inhibited the protective effects of SIRT3 overexpression.

**Conclusion:**

It is the first time that we confirm CVB-D ameliorating cardiomyocyte senescence in DCM by enhancing mitochondria dysfunction through activated SIRT3–ATP5O axis. It also suggests that CVB-D could be employed in the future to treat cardiomyocyte senescence caused by DCM.

**Graphical Abstract:**

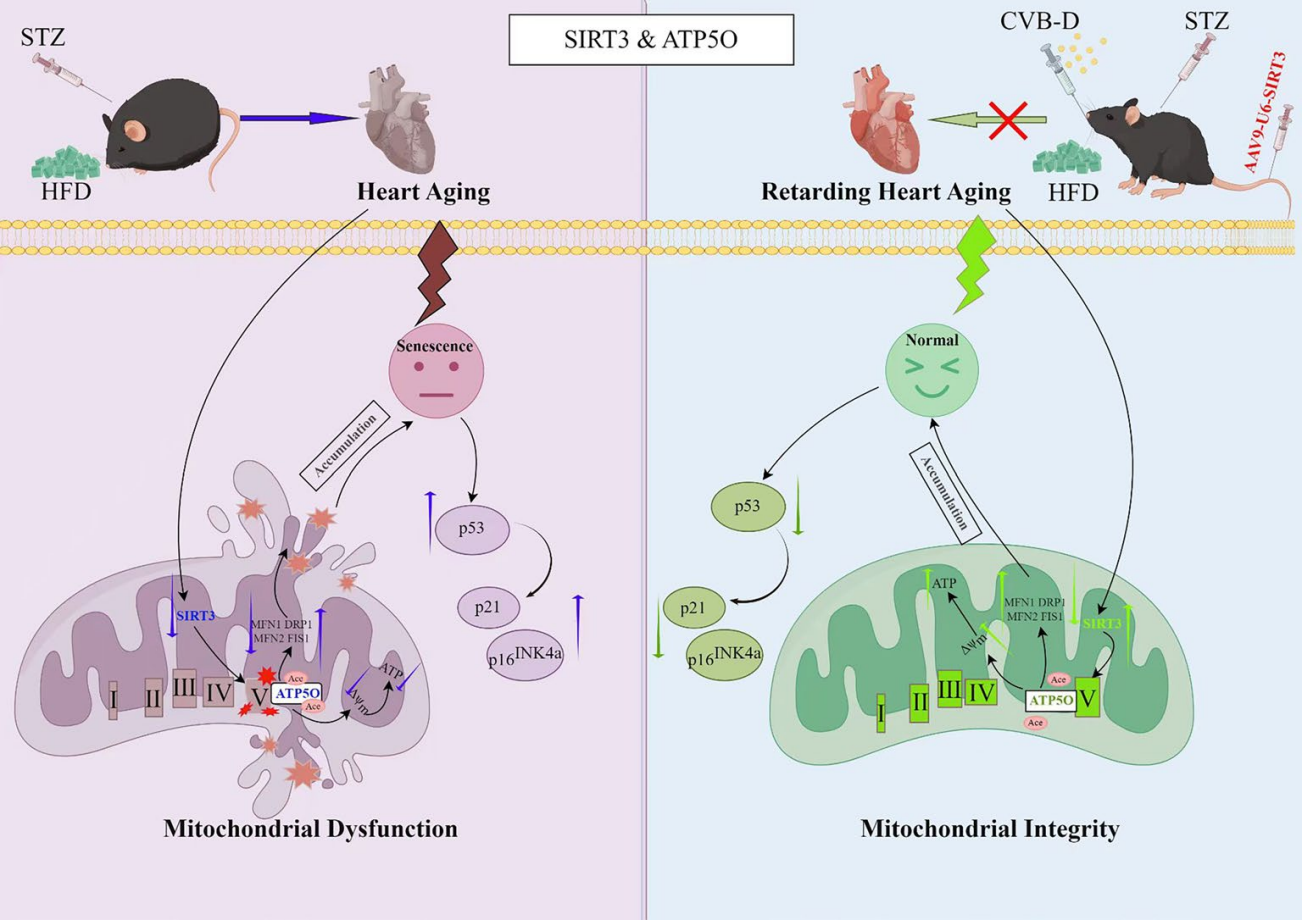

**Supplementary Information:**

The online version contains supplementary material available at 10.1186/s13020-025-01254-3.

## Introduction

In 2021, around 537 million people globally were affected by diabetes, and this figure is expected to rise to 600 million by the end of the decade. Type 2 diabetes mellitus (T2DM), which makes up 90–95% of total diabetes cases [1].

Diabetic cardiomyopathy (DCM), a serious cardiovascular disorder caused by diabetes, has seen a rising prevalence worldwide in recent years [2]. Cumulative evidence indicates DCM represents an independent leading cause of mortality in type 2 diabetes patients [3, 4]. Despite its clinical importance, the precise molecular mechanisms underlying DCM remain incompletely understood, resulting in limited targeted therapeutic options, identifying novel therapeutic targets is critical.

DCM and cardiomyocyte senescence share significant pathological overlap in molecular mechanisms, providing new insights into the pathogenesis of DCM [5]. Cardiomyocyte senescence has emerged as a key contributor to age-related cardiac dysfunction [6]. Cardiomyocyte senescence represents an independent risk factor for cardiovascular diseases [7]. Mitochondria undergo significant remodeling during cardiomyocyte senescence [8]. Mitochondrial dysfunction represents a cardinal feature of cardiomyocyte senescence. Dysregulated mitochondrial fission–fusion dynamics exacerbate the senescent phenotype in cardiomyocytes [9].

Notably, the maintenance of mitochondrial function is closely linked to the sirtuin family of proteins [10]. Sirtuin 3 (SIRT3) functions as an NAD + -dependent mitochondrial deacetylase, maintaining mitochondrial function and reducing oxidative stress through deacetylation [11]. Research indicates that SIRT3 can regulate lifespan and deacetylate ATP5O (ATP synthase subunit O) [12]. Moreover, studies indicate that reduced SIRT3 activity leads to abnormal accumulation of acetylated proteins, impairing oxidative phosphorylation (OXPHOS), increasing ROS production, and reducing mitochondrial membrane potential, thereby accelerating cardiomyocyte senescence [13].

ATP5O (ATP synthase subunit O), a key component of the mitochondrial F1F0 ATP synthase (Complex V), regulates enzyme rotational activity to ensure efficient ATP production and maintain structural integrity of the F1F0 complex [14]. Concurrently, reduced ATP5O expression has been linked to shortened lifespan, mitochondrial dysfunction [15], increased protein acetylation, altered mitochondrial dynamics, and accelerated aging disease progression [16]. However, in the context of cardiomyocyte senescence, the mechanistic role of the SIRT3–ATP5O axis remains unclear. Whether SIRT3–ATP5O participates in regulating cardiomyocyte senescence and whether it could serve as a novel therapeutic target for aging-related cardiac diseases are pressing questions that require further investigation.

Boxwood, scientifically classified as *Buxus microphylla* Sieb. et Zucc. var. *sinica* Rehd. et Wils., is a medicinal plant whose stems, branches, and leaves are used in traditional medicine. Phytochemical studies have identified Cyclovirobuxine D (CVB-D, C₂₆H₄₆N₂O), as the primary bioactive component of boxwood [17]. Cyclovirobuxine D has been developed into a pharmaceutical drug named Huangyangning [18]. Recent research has revealed that CVB-D can ameliorate dilated cardiomyopathy (DCM) by improving SIRT3-mediated mitochondrial function [19]. Additionally, CVB-D helps maintain mitochondrial dynamics by inhibiting excessive fission and promoting fusion, thereby preserving mitochondrial network integrity [20]. However, the role of CVB-D in suppressing DCM-associated cardiomyocyte senescence remains incompletely understood. This study aimed to construct in vivo and in vitro models of DCM-related cardiomyocyte senescence to investigate the pharmacological mechanisms by which CVB-D modulates SIRT3–ATP5O-mediated mitochondrial function in DCM.

## Materials and methods

### Reagents

Cyclovirobuxine D (CVB-D, C₂₆H₄₆N₂O) was purchased from Chengdu Pusi Biotechnology Co., Ltd., with catalog number PS1540, batch number PS020619, and a drug purity of 98%. HFD comprising 60 kcal% fat was purchased from Moldiets Biotechnology Inc. (M10160). GPU6-ATP5O and GCMV-SIRT3 were purchased from Shanghai GenePharma Biotechnology Co., Ltd., with the following sequences: sense 5′-CGGCCAACCTCATGAATTTAC-3′ and NM_001127351.1. The adeno-associated virus 9 vectors for SIRT3 shRNA (AAV9-U6-SIRT3) (5′-CTGAGTCCTCGAAGGAAAGAT-3′) and its negative control (AAV9-U6-NC) (5′-ACTACCGTTGTTATAGGTG-3′) were purchased from Shanghai GenePharma Biotechnology Co., Ltd.

### Animals

Male C57BL/6J mice (6–8 weeks old, 18–24 g) were purchased from the Experimental Animal Center of Guizhou Medical University.

Following a 1-week acclimation period, male C57BL/6J mice were randomly assigned to two groups: normal diet (Control) and high-fat diet (HFD). DCM group mice received intraperitoneal injections of streptozotocin (STZ, 45 mg/kg/day) for four consecutive days to induce diabetes. Mice with fasting blood glucose ≥ 11.1 mmol/L were confirmed as diabetic models. Diabetic mice were equally randomized into four groups: HFD (DCM), HFD + low-dose CVB-D (0.5 mg/kg/day, CVB-D-L), HFD + high-dose CVB-D (1 mg/kg/day, CVB-D-H), and HFD + metformin (250 mg/kg/day, Met). CVB-D and metformin were administered via daily gastric gavage for 8 weeks. The control and DCM groups received daily saline injections. The mice were euthanized under isoflurane anesthesia for subsequent experiments.

### Echocardiographic function

The mice were anesthetized via inhalation of 1–2% isoflurane and positioned on a heated table with continuous low-dose anesthesia maintained throughout the procedure. M-mode echocardiography was conducted by blinded operators via a VINNO6LAB ultrasound system (VINNO, Suzhou, China) with a 10 MHz probe to assess cardiac function.

### Histological staining

Hearts were harvested transversely and fixed in 4% paraformaldehyde for 24 h. The tissues were then dehydration, paraffin embedded, sectioned and stained. The sections were dehydrated and mounted. The stained sections were visualized via a Nikon Eclipse E100 light microscope (Tokyo, Japan) and quantified via ImageJ software (NIH, Bethesda, MD, USA).

### Ultrastructural examination of the myocardium

Fresh left ventricular tissue was cut into 1 mm^3^ pieces and fixed sequentially in 3% glutaraldehyde and 1% osmium tetroxide. The samples were processed through serial acetone dehydration, impregnation with epoxy resin 812, and embedding. Semithin sections were stained with methylene blue, while ultrathin sections were cut with diamond knives and double-stained with uranyl acetate and lead citrate. The sections were visualized via a JEM-1400-FLASH transmission electron microscope.

### Separation and purification of primary mouse cardiomyocytes

C57BL/6 J mice (18–22 g, SPF grade) were procured from Beijing Biotechnology Co., Ltd. Primary neonatal mouse ventricular myocytes (NMVMs) were isolated and purified from 1–3-day-old neonatal mice and cultured in DMEM at 37 °C in a 5% CO_2_ incubator. Cardiomyocytes (10^5^ cells/mL) were treated with 100 μM 5-BrdU to suppress cardiac fibroblast proliferation.

### HL-1 cell culture and treatment

HL-1 cardiomyocytes (Zishan Technology, Changsha, China) were used to assess cell cycle distribution. High glucose/palmitic acid (HG/PA) culture conditions were used to mimic diabetic cardiomyopathy (DCM). The cell cycle distribution of HL-1 cells treated with HG/PA, CVB-D, or metformin was analyzed by flow cytometry.

### Immunofluorescence analysis

Neonatal mouse ventricular myocytes (NMVMs) were fixed onto glass coverslips. The cells were permeabilized with 0.2% Triton X-100 for 20 min, followed by blocking with 5% bovine serum albumin for 90 min at room temperature. After each step, cells were washed 3 × with PBS. The cells were incubated with primary antibodies against cTnT (1:500 dilution), p53 (1:200), SIRT3 (1:100), p21 (1:400), MFN1 (1:100), MFN2 (1:100), p16 (1:500), DRP1 (1:100), FIS1 (1:100), and ATP5O (1:50). Fluorescent secondary antibodies (anti-rabbit or anti-mouse) were applied for 90 min at room temperature. The cells were counterstained with DAPI. Fluorescence micrographs were acquired via a Nikon Eclipse C1 fluorescence microscope (Tokyo, Japan) or Olympus FV1000 confocal microscope. Images were analyzed via ImageJ software (Version 1.52a, NIH, USA).

### Western blotting analysis

Myocardial tissues and NMVMs were rinsed three times with precooled PBS and then to extract protein. The protein concentration was quantified via a BCA kit, and samples were denatured in loading buffer at 100 °C for 5–7 min. The proteins were separated via 0.22-μm PVDF membrane. The membranes were blocked with 5% skim milk in 1% TBS-Tween-20 buffer for 2 h at room temperature. The membranes were incubated overnight at 4 °C with primary antibodies: SIRT3 (ab246522, Abcam), p53 (ab246522), p21 (ab246522), p16 (ab246522), MFN1 (ab246522), FIS1 (ab246522), and GAPDH (ab10000). Following washes, membranes were incubated with HRP-conjugated secondary antibodies for 90 min at room temperature. The membranes were visualized via an NcmECL High Kit (NCM Biotech, Suzhou, China). Protein levels were normalized to GAPDH. Band intensity was quantified via Image Lab software (Bio-Rad, v5.2) and ImageJ (Version 1.52a, NIH, USA).

### Cellular thermal shift assay (CETSA)

NMVMs were pretreated with 0.5 μM CVB-D or equivalent DMSO for 48 h and then lysed with RIPA buffer to extract total protein. After protein quantification via a commercial BCA kit, samples were divided into nine aliquots and heated from 50 °C to 90 °C for 5 min via a thermal cycler. Thermal stability was analyzed via western blotting [21].

### Flow cytometry

The cell cycle distribution of HL-1 cells was analyzed via a cell cycle detection kit (Beyotime, Shanghai, China). The collected HL-1 cells were centrifuged and fixed with 500 μL of 70% prechilled ethanol at 4 °C overnight. Treated cells were resuspended in staining solution (propidium iodide:RNase A = 9:1) and incubated in the dark for 30 min. Intracellular ROS levels were measured via DCFH-DA staining and a reactive oxygen species assay kit (Beyotime, Shanghai, China). Flow cytometry analysis was performed using an ACEA NovoCyte flow cytometer (ACEA Biosciences, Hangzhou, China).

### Measurement of oxygen consumption rate (OCR)

Mitochondrial function in treated NMVMs was assessed via oxygen consumption rate (OCR) measurements via an Oroboros O2K respirometer. Following baseline respiration measurement, sequential addition of mitochondrial inhibitors—oligomycin (1 μM), carbonyl cyanide 4-(trifluoromethoxy) phenylhydrazone (2 μM), and rotenone/antimycin A (1 μM each)—was performed to determine spare respiratory capacity, maximal respiration, and ATP production, respectively.

### Mitochondrial membrane potential (ΔΨm) assay

The ΔΨm was assessed via a JC-1 staining kit (Solarbio, Cat. M8650, Beijing, China). Following drug treatment, NMVMs were washed and incubated with JC-1 working solution at 37 °C for 20 min. After staining, cells were washed twice with JC-1 staining buffer. JC-1 exists in two distinct fluorescence states: monomeric form (green fluorescence, 530 nm) and J-aggregates (red fluorescence, 590 nm). Fluorescence images were acquired via a Nikon Eclipse C1 microscope (Tokyo, Japan). The ΔΨm was quantified via ImageJ software.

### Senescence-contacted β-galactosidase (SA-β-gal) dyeing

Senescence-associated β-galactosidase (SA-β-gal) staining was performed via a commercial kit to assess senescence levels in NMVMs following the manufacturer's protocol. NMVMs were harvested from culture medium, washed with ice-cold PBS, fixed in β-Gal solution at room temperature for 15 min, rinsed, and incubated overnight at 37 °C with staining solution (containing Mix Reagents B, C, D, and E at a 5:1:1:93 ratio). Images were acquired via bright-field microscopy (Nikon Eclipse C1, Tokyo, Japan). The percentage of SA-β-gal-positive cells was quantified via ImageJ software (v1.52a).

### Measurement of mitochondrial complex V activity

Mitochondrial complex V activity was measured via a complex V/ATP synthase activity assay kit (Solarbio, China; Cat. No. BC3975) according to the manufacturer’s protocol. Mitochondrial homogenates from NMVMs or cardiac tissues were prepared in assay buffer. Complex V activity was quantified via a Thermo Fisher Scientific microplate reader (Waltham, MA, USA). Complex V activity was expressed as nmol/min/mg protein.

### Molecular docking

The 3D structure files of SIRT3-MOUSE (UniProt ID: Q8R104) and ATP-MOUSE (UniProt ID: Q9DB20) predicted by Alphafold were downloaded via SIRT3 and ATP5O as keywords from UniProt (https://www.uniprot.org/). We employed Schrodinger software (Schrödinger Maestro v13.5) to prepare the proteins. The best binding pocket was predicted by Sitemap, and the docking parameters were set for Glide docking, which yielded semiflexible docking results for the small protein molecule SIRT3-MOUSE with CVB-D. The semiflexible docking results were flexibly optimized by Schrödinger IFD (Induced Fit Docking) to obtain the complex of SIRT3-MOUSE with CVB-D. PyMOL (PyMOL v2.5.4) was used to draw 3D interactions. Using HADDOCK 2.4 (https://wenmr.science.uu.nl/haddock2.4/), the flexibly optimized complex of SIRT3-MOUSE with CVB-D was docked with ATP5O-MOUSE. We optimized the docking results with Rosetta Docking (https://www.rosettacommons.org/) flexibility and then employed Schrodinger Glide for redocking to obtain the final PLP (Protein–Liagand-Protein) complex.

### Coimmunoprecipitation (Co-IP)

Total protein was extracted from lysis buffer and incubated with primary antibodies against ATP5O (1:50), SIRT3 (1:50), or acetylated lysine (1:50) overnight at 4 °C. Protein A/G-agarose beads (Sangon Biotechnology, Shanghai, China) were added to form protein complexes, which were gently mixed overnight at 4 °C for immunoprecipitation. Immunoprecipitated complexes were analyzed via western blotting with anti-SIRT3 (1:1000, Abcam) and anti-ATP5O (1:1000, Cell Signaling Technology) antibodies.

### Microscale thermophoresis (MST)

MST is an emerging biomolecular interaction analysis technology developed on the principle of thermal phoresis (microscale thermophoresis). The binding affinity of CVB-D to SIRT3 was determined by MST, and a constant KD value was obtained by analyzing the results with the MO.Affinity Analysis (NanoTemper Technologies).

### Surface plasmon resonance (SPR) binding assay

Surface plasmon resonance (SPR) was used to evaluate protein–protein interactions between murine SIRT3 and ATP5O. All procedures were performed according to a previously described protocol [22]. Following standard procedures, a carboxyl sensor chip was installed on the SPR instrument. The target SIRT3 protein was immobilized onto a carboxyl sensor chip. ATP5O protein was diluted to varying concentrations in running buffer and sequentially injected from low to high concentrations. The kinetic parameters of binding interactions were analyzed via Trace Drawer software (Biacore, GE Healthcare). Both SIRT3 (KBS2024022702) and ATP5O (KBS2024022701) recombinant proteins exhibited purities exceeding 90%.

### Isothermal Titration Calorimetry (ITC)

The interaction between SIRT3 and ATP5O was analyzed via a Nano ITC (isothermal titration calorimeter; TA Instruments, New Castle, DE, USA). Briefly, ATP5O protein was titrated into a SIRT3-containing solution at 25 °C, and heat released during binding was monitored in real time. Binding curves were generated via data analysis via NanoAnalyze software (TA Instruments).

### LC–MS/MS analysis

LC–MS/MS analysis was performed via an Orbitrap Elite mass spectrometer (Thermo Fisher Scientific, Waltham, MA, USA). Cardiac tissues from each group were prepared as previously described (Guan C et al., 2021). The data were analyzed via Proteome Discoverer 1.4 (Thermo Fisher Scientific), with the *UniProtKB-Bifidobacterium bifidum ATCC 29521.fasta* database (*UniProt accession UP000008227*) selected for peptide identification.

### Short RNA (shRNA) transfection

SIRT3 shRNA (GenePharma, Shanghai, China) was transfected into cells via Lipofectamine 2000 (Invitrogen, Carlsbad, CA, USA). SIRT3 shRNA sequences were: sense 5’-CTGAGTCCTCGAAGGAAAGATTTCAAGAGAATCTTTCCTTCGAGGACTCAGTT-3’ and negative control 5’-TTCTCCGAACGTGTCACGTTTCAAGAGAACGTGACACGTTCGGAGAATT-3’. Transfection efficiency was validated by Western blotting, immunofluorescence, and qPCR.

### Mitochondrial damage five-level scoring system

The mitochondrial damage five-level scoring system is a semi-quantitative morphological assessment technique. In this method, researchers examine a specified number of mitochondria, typically 100, using an electron microscope. They then evaluate and score these mitochondria based on the percentage exhibiting pathological features, such as swelling or cristae rupture. The scoring is as follows: 0 points for less than 5% affected, 1 point for 5%-25% affected, 2 points for 25%-50% affected, 3 points for 50%-75% affected, and 4 points for more than 75% affected. This approach facilitates a standardized assessment and enables inter-group comparisons regarding the extent of mitochondrial damage.

### Statistical analysis

All the data were subjected to analysis via GraphPad Prism 8.0 software. One-way ANOVA was used for multigroup comparisons, whereas Student’s *t* test was used for comparisons between two groups. All statistically analyzed outcomes are presented as the mean ± SEM. A *p* value < 0.05 indicates a statistically significant difference in this study.

## Results

### CVB-D alleviates DCM progression in diabetic mice

**Fig. 1:**
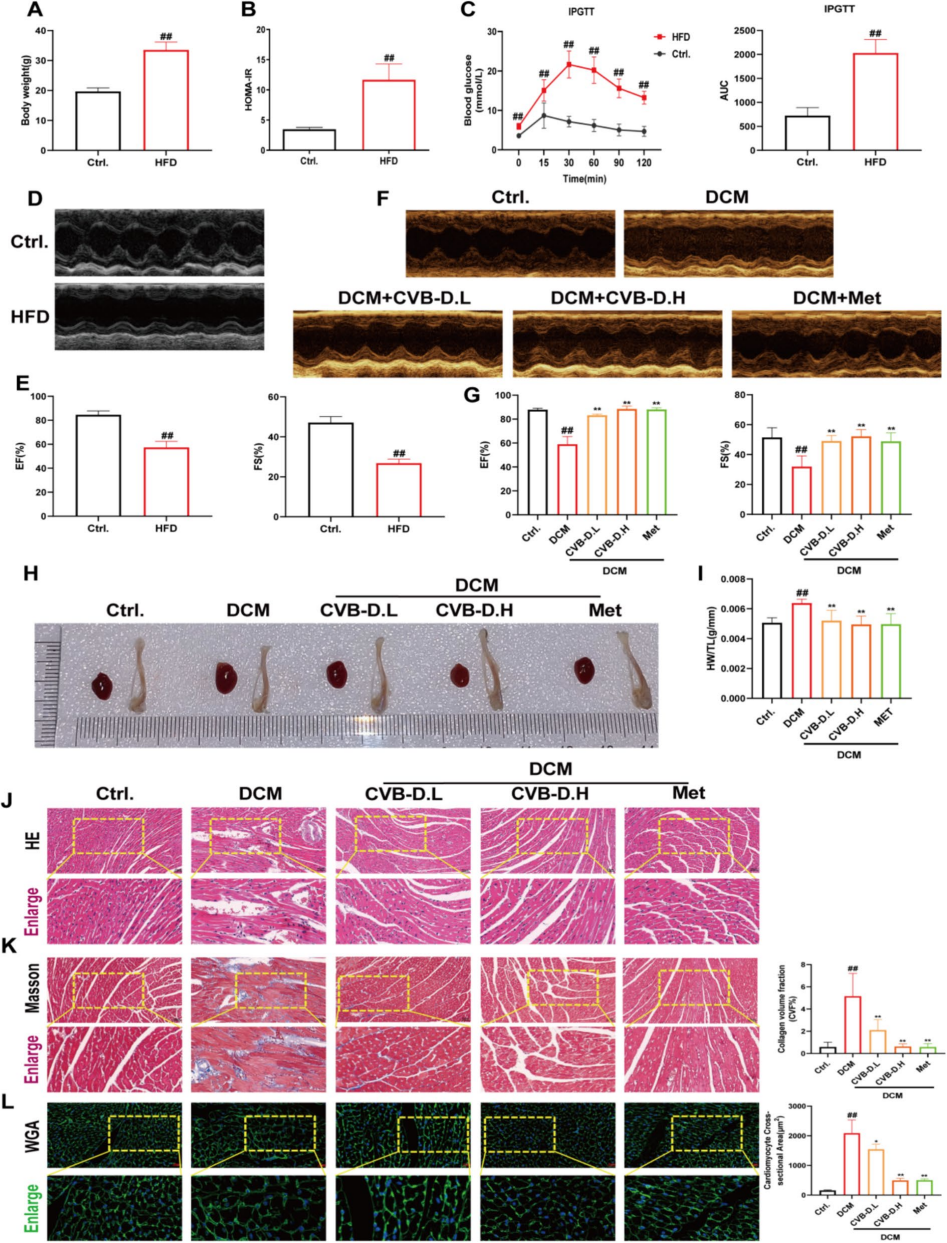
CVB-D ameliorated HFD combined with streptozotocin (STZ)-induced DCM in mice. **A** Body weights of the mice (n = 6). **B** Insulin resistance indices of the mice (n = 6). **C** Oral glucose tolerance test results for different groups and the area under the curve (AUC) of blood glucose in mice (n = 6). **D**–**G** Representative M-mode echocardiographic images and data analysis of the EF% and FS% (n = 6). **H** Anatomical images of the mouse heart and tibia (n = 6). **I** Calculation of heart weight/tibia length (HW/TL) (n = 6). **J** Heart tissue samples were dyed with H&E (HE) (scale bar = 25 µm). **K** Masson’s trichrome staining of collagen deposition heart tissue (scale bar = 25 µm) and quantitative analysis of collagen deposition (n = 6). **L** WGA dyeing assay of cardiomyocytes in the DCM (scale bar = 25 µm) and quantitative analysis of the cross-sectional area of cardiomyocytes (n = 6). ^*#*^*p* < 0.05, ^*##*^*p* < 0.01 versus the control group; ^*^p < 0.05, ^**^p < 0.01 versus the model group

T2DM mouse model was induced via HFD feeding combined with intraperitoneal STZ injections (Graphical timeline). After 12 weeks of model induction, significant differences in body weight and insulin resistance were observed between HFD and control groups (Fig. 1A and B). Moreover, HFD-fed mice presented significantly elevated blood glucose levels and areas under the curve (AUCs) during oral glucose tolerance test (OGTTs), reflecting impaired glucose tolerance (Fig. 1C). Echocardiography revealed significantly greater ejection fraction (EF%) and fractional shortening (FS%) in control mice than in HFD-induced DCM mice, confirming impaired cardiac function in the DCM mice(Fig. 1D and E). CVB-D at different concentrations (0.5 or 1 mg/kg/d) and metformin (Met, 250 mg/kg/d) were administered via oral gavage. Echocardiography showed significantly reduced EF% and FS% in DCM mice compared to controls. Treatment with CVB-D (0.5 or 1 mg/kg/d) or metformin (Met, 250 mg/kg/d) significantly improved diastolic function in DCM mice (Fig. 1F and G). Representative images of hearts and tibias showed significantly increased heart weight-to-tibia length ratio (HW/TL) in DCM mice, which was attenuated by CVB-D treatment (Fig. 1H and I). Serum analysis revealed significantly decreased high-density lipoprotein (HDL) and increased triglyceride (TG), total cholesterol (TC), and low-density lipoprotein (LDL) in DCM mice. These lipid abnormalities were significantly reversed by CVB-D treatment (**Fig. S1**). Histopathological analysis was performed to evaluate the therapeutic effects of CVB-D on DCM. Hematoxylin‒eosin (H&E) and wheat germ agglutinin (WGA) staining revealed enlarged cardiomyocyte nuclei, hypertrophied cardiomyocytes, and disorganized myocardial fibers in DCM mice compared with controls. CVB-D significantly ameliorated cardiac remodeling. Masson’s trichrome staining revealed significantly increased myocardial collagen content in DCM mice, which was significantly reduced by CVB-D (Fig. 1J, K and L). Collectively, these results demonstrate that CVB-D effectively ameliorates pathological features of DCM. In conclusion, CVB-D effectively retards the progression of DCM in mice.

**Figure:**
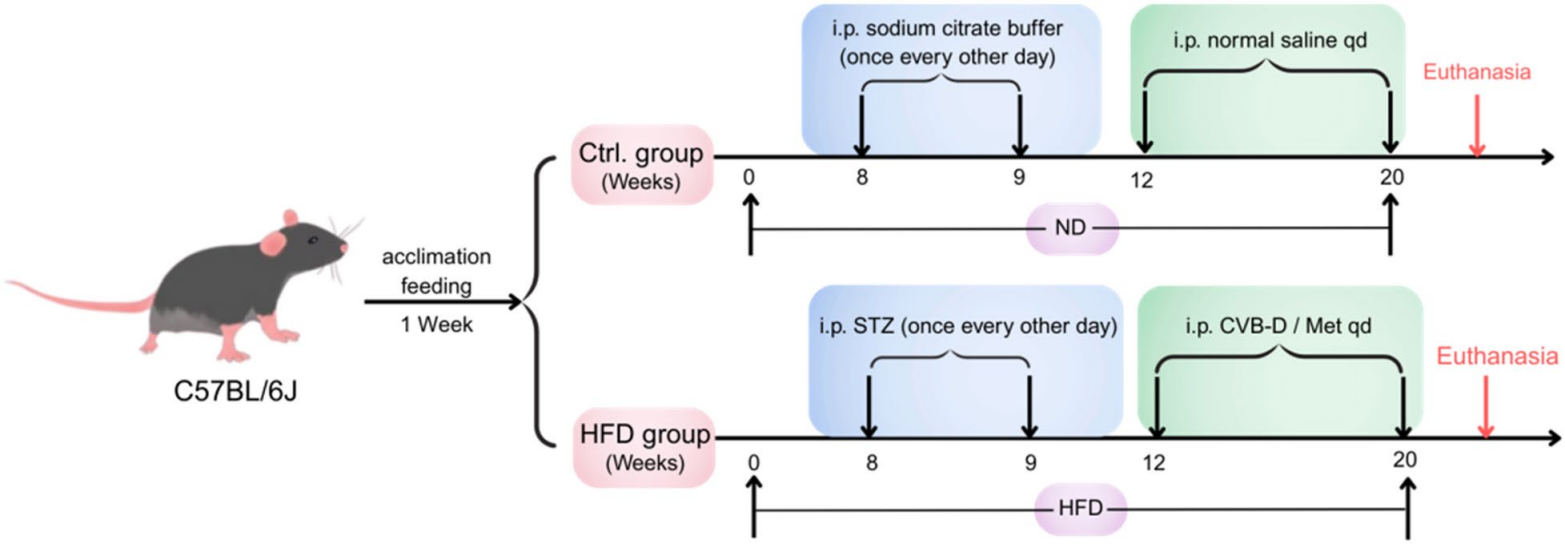
Graphical timeline

### CVB-D improved mitochondrial function in DCM mice

**Fig. 2:**
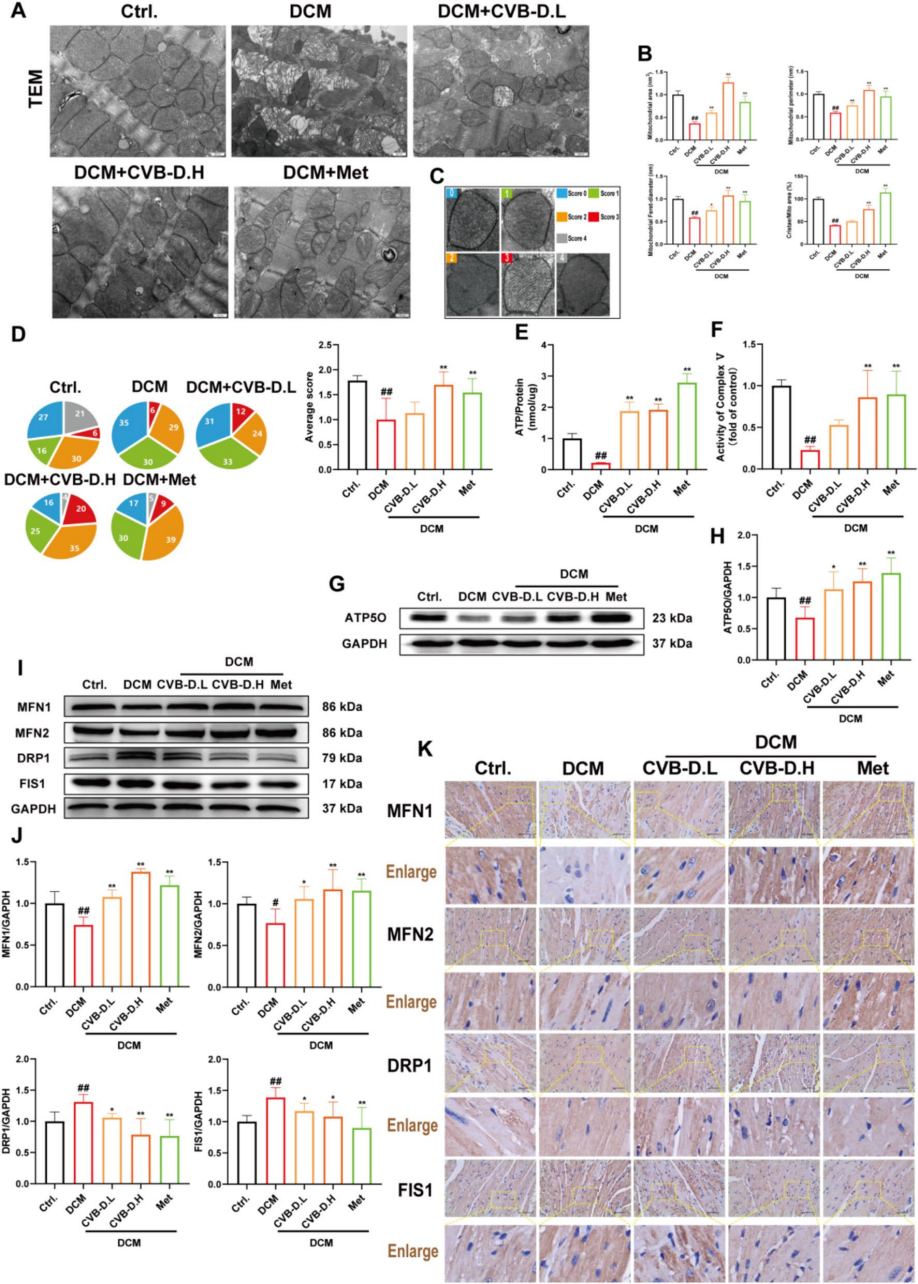
CVB-D ameliorates mitochondrial dysfunction in DCM mice. **A** Representative transmission electron microscopy (TEM) images of mitochondria in heart tissue (scale bar = 500 nm). **B** Quantification of average mitochondrial cross-sectional area (nm^2^), mitochondrial perimeter (nm), mitochondrial Feret diameter (nm), and ratio of the crista area to mitochondrial area (%) in heart tissue via ImageJ (n = 6). **C** A representative mitochondrial appearance was selected and scored by a 5-level scoring system. **D** Pie chart showing the score distribution of the mitochondria in each group, and the bar graph shows the mean mitochondrial score for each group (70–120 mitochondria were randomly selected for each group). **E** ATP content of mouse cardiac tissues (n = 3). **F** Activity of ATP synthase in mouse cardiac tissues (n = 3). **G** and **H** ATP5O were analyzed by Western blotting (n = 5). **I** and **J** Western blotting analysis of MFN1, MFN2, DRP1, and FIS1 protein levels in mouse cardiac tissues (n = 5). **K** Representative images of immunohistochemical staining for MFN1, MFN2, DRP1, and FIS1 in heart tissue (scale bar = 40 µm). ^*#*^*p* < 0.05, ^*##*^*p* < 0.01 versus the control group; ^*^p < 0.05, ^**^p < 0.01 versus the model group

Transmission electron microscopy (TEM) revealed significantly reduced mitochondrial cross-sectional area, perimeter, Feret diameter, and crista area-to-matrix area ratio in DCM mice, which were partially restored by CVB-D treatment (Fig. 2A and 2B). Mitochondrial morphology was evaluated using a 5-level scoring system [35], with significantly lower scores observed in DCM mice compared to controls. This phenotype was partially rescued by CVB-D (Fig. 2C and D). Mitochondria are the primary site of ATP synthesis. To evaluate mitochondrial function, ATP content and ATP synthase activity were measured in cardiac tissues. Long-term HFD feeding significantly reduced ATP levels and ATP synthase activity, which were reversed by CVB-D (Fig. 2E and F). ATP5O is a critical subunit of mitochondrial ATP synthase localized to the mitochondrial matrix. Mitochondrial dynamics (fusion/fission) play a key role in DCM pathogenesis. The expression of key regulators of mitochondrial dynamics, including MFN1, MFN2, DRP1, and FIS1, is altered in DCM. Western blotting was used to assess ATP5O expression in DCM mice treated with CVB-D. ATP5O expression was significantly downregulated in DCM mice but restored by CVB-D treatment (Fig. 2G and H). To investigate the protective effects of CVB-D on mitochondrial dynamics, western blotting was performed to analyze key fusion/fission proteins. DCM mice presented significantly reduced MFN1/MFN2 (fusion markers) and increased DRP1/FIS1 (fission markers). These changes were reversed by CVB-D treatment (Fig. 2I and J). Immunohistochemical staining confirmed western blot results, which revealed restored MFN1/MFN2 and reduced DRP1/FIS1 expression in DCM-treated mice (Fig. 2K). Oxidative stress contributes to mitochondrial dysfunction in DCM. Compared with those in control mice, dihydroethidium (DHE) staining revealed significantly increased ROS levels in DCM mice. These effects were reversed by CVB-D treatment, which reduced ROS levels (**Fig. S2**).

### CVB-D ameliorates mitochondrial dysfunction in HG/PA-induced primary mouse cardiomyocytes

**Fig. 3:**
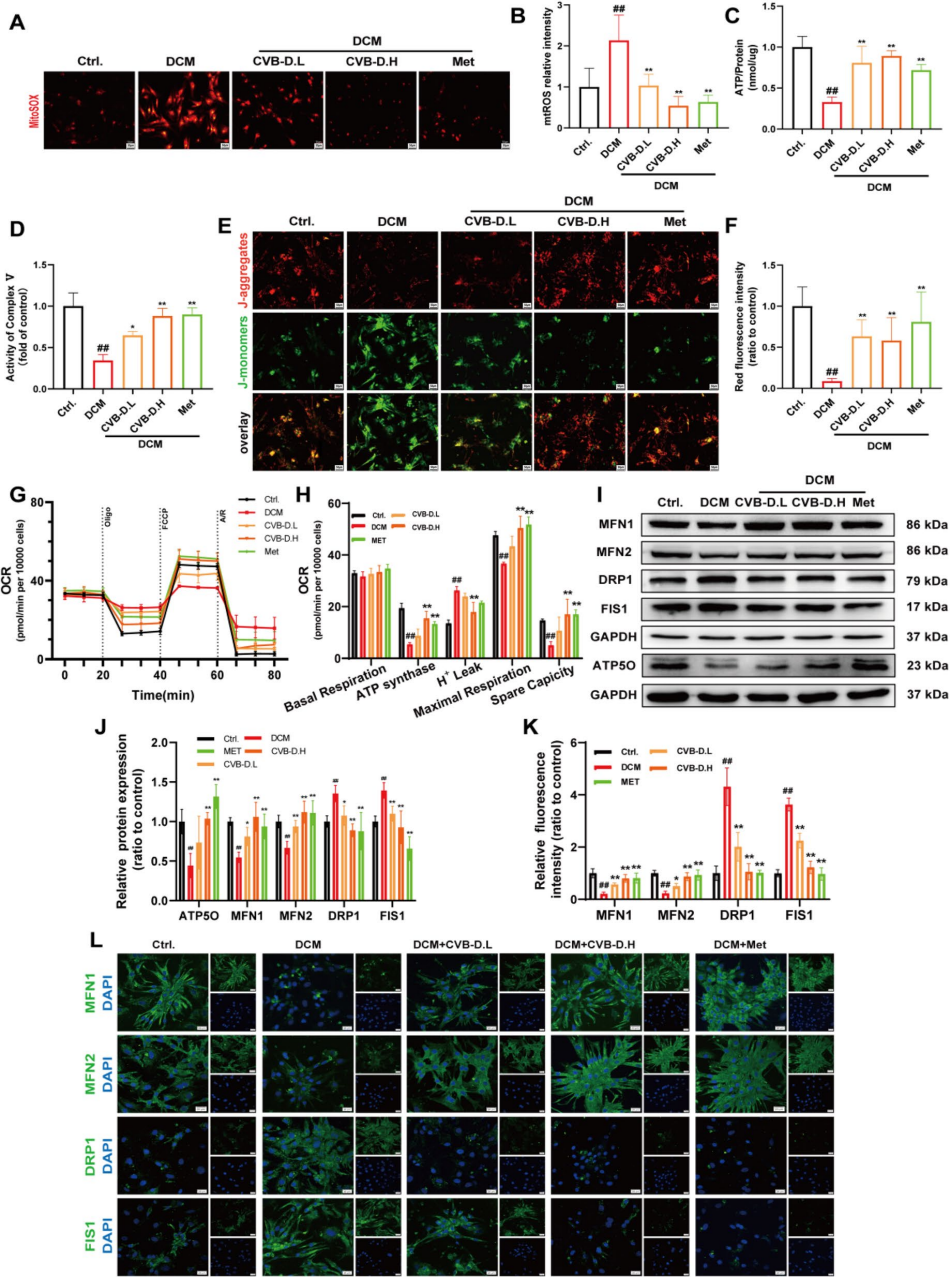
CVB-D ameliorates HG/PA-induced mitochondrial dysfunction in NMVMs. **A** MitoSOX fluorescence staining (scale bar = 50 µm). **B** Quantification of MitoSOX fluorescence in NMVMs via ImageJ (n = 5, scale bar = 50 µm). **C** ATP content of NMVMs (n = 3). **D** Activity of ATP synthase in NMVMs (n = 3). **E** Representative images of JC-1 staining (scale bar = 50 µm). **F** Quantification of red fluorescence measuring in JC-1 dyeing via ImageJ (n = 5). **G** and **H** The OCR was measured via the Oroboros O2K assay (n = 3). **I** and **J** Western blotting analysis of ATP5O, MFN1, MFN2, DRP1, and FIS1 protein levels in NMVMs (n = 5). **K** Immunofluorescence statistical analysis (n = 5). **L** Immunofluorescence images of MFN1, MFN2, DRP1, and FIS1 in NMVMs (scale bar = 20 µm). ^*#*^*p* < 0.05, ^*##*^*p* < 0.01 versus the control group; ^*^p < 0.05, ^**^p < 0.01 versus the model group

Neonatal mouse ventricular myocytes (NMVMs) were isolated via trypsin digestion and characterized via immunofluorescence (IF) staining. Cardiac troponin T (cTnT) immunostaining confirmed the identity of the cardiomyocytes (Fig. S3A). HG/PA-induced NMVMs were used to evaluate the protective effects of CVB-D. MTT assays revealed that 40 mM HG + 200 μM PA treatment for 48 h significantly reduced NMVM viability (Fig. S3 B), which was rescued by 2 h pretreatment with CVB-D (0.1 μM) or CVB-D (0.5 μM) (Fig. S3 C). LDH release, a marker of cell injury, was significantly lower in CVB-D-pretreated NMVM group than in the DCM group (Fig. S3D). Mitochondrial function is critical for cardiomyocyte homeostasis. Mito-SOX staining was used to assess mitochondrial oxidative stress in NMVMs. Compared with those of the controls, fluorescence imaging revealed significant mitochondrial ROS accumulation in DCM-induced NMVMs. These abnormalities were significantly ameliorated by CVB-D treatment (Fig. 3A and B). Consistent with in vivo findings, CVB-D increased ATP content and ATP synthase activity in HG/PA-treated NMVMs (Fig. 3C and D). JC-1 staining revealed significantly reduced mitochondrial membrane potential (ΔΨm) in DCM-induced NMVMs, which was restored by CVB-D (Fig. 3E and F). Oroboros O2K respirometry revealed that DCM-induced NMVMs presented reduced ATP-linked respiration, maximal respiration, and spare respiratory capacity, along with increased proton leakage. These bioenergetic deficits were reversed by CVB-D treatment (Fig. 3G and H). Western blot analyses confirmed that CVB-D reversed DCM-induced downregulation of ATP5O in NMVMs (Fig. 3I and J). To further investigate the mechanism, Western blot analysis was performed to assess mitochondrial dynamics-related proteins (MFN1, MFN2, DRP1, FIS1) in HG/PA-treated NMVMs. CVB-D pretreatment revealed MFN1/MFN2 downregulation and DRP1/FIS1 upregulation in HG/PA-treated NMVMs (Fig. 3I and J). IF staining corroborated the Western blot findings, revealing restored mitochondrial dynamics marker (MFN1, MFN2, DRP1, FIS1) expression in CVB-D-treated NMVMs (Fig. 3K and L). Oxidative stress was similarly dysregulated in DCM-induced NMVMs, as DCFH-DA staining and flow cytometry revealed reduced ROS accumulation in CVB-D-treated cells (**Fig. S4 A and B**).

### CVB-D inhibits senescence and upregulates the expression of SIRT3

**Fig. 4:**
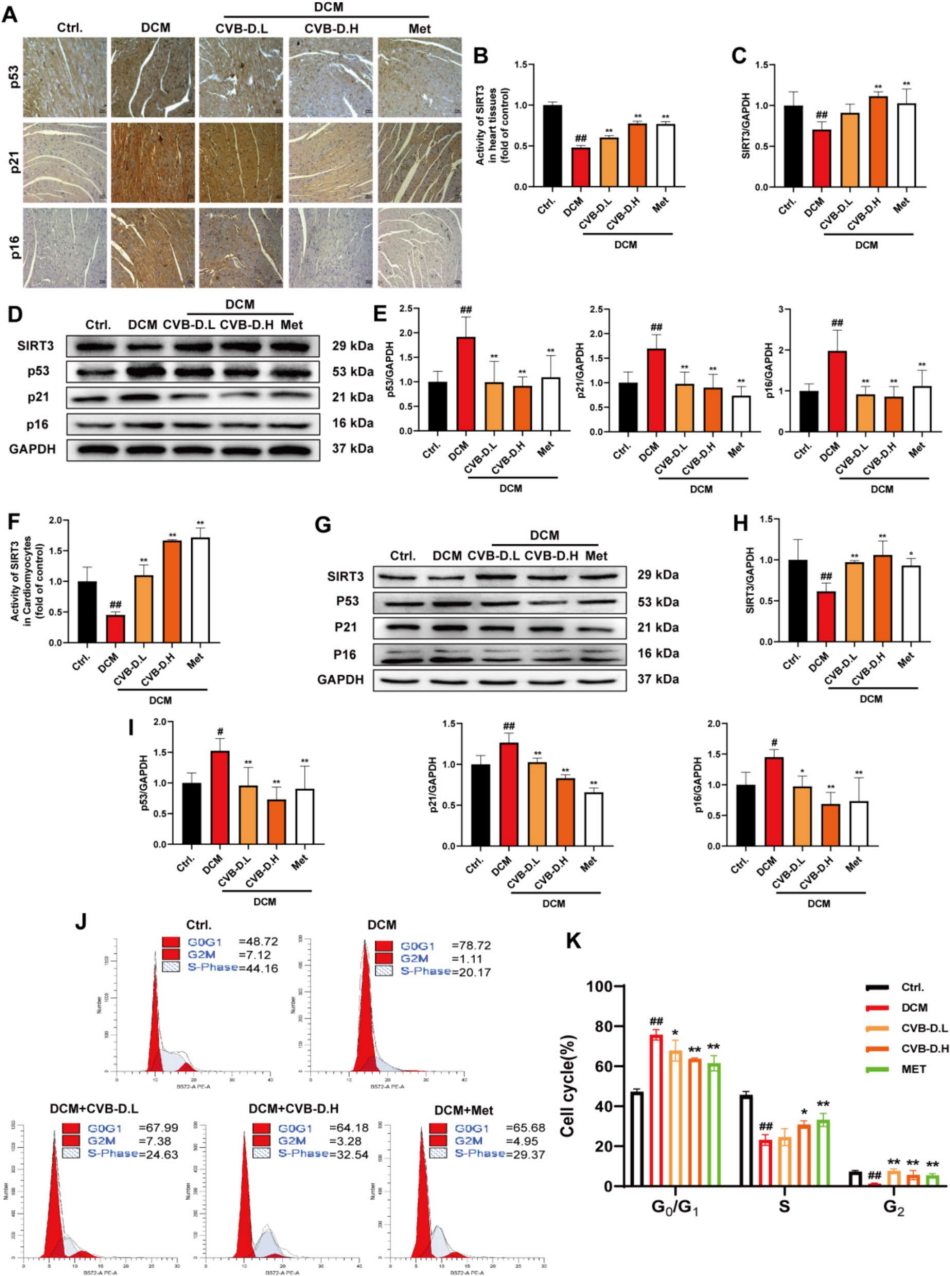
CVB-D upregulates the expression of SIRT3 and alleviates senescence. **A** Immunohistochemical images of p53, p21, and p16 in DCM mice (scale bar = 25 µm). **B** Activity of SIRT3 in DCM mice (n = 3). **C**, **D** and **E** Western blotting analysis of SIRT3, p53, p21, and p16 protein levels in DCM mice (n = 5). **F** Activity of SIRT3 in NMVMs (n = 3). **G**, **H** and **I** Western blotting analysis of SIRT3, p53, p21, and p16 protein levels in NMVMs (n = 5). **J** Flow cytometry was used to examine the cell cycle distribution of HL-1 cells. **K** Quantitative analysis of flow cytometry (n = 3). ^*#*^*p* < 0.05, ^*##*^*p* < 0.01 versus the control group; ^*^p < 0.05, ^**^p < 0.01 versus the model group

We conducted immunohistochemical staining and western blotting on mouse heart tissues to confirm the role of CVB-D in delaying cardiac senescence and to detect senescence-related changes, respectively. We examined the levels of senescence-related biomarkers via immunohistochemistry (Fig. 4A). SIRT3 activity measurements revealed that SIRT3 expression was significantly downregulated in the DCM group. CVB-D effectively increased SIRT3 activity (Fig. 4B). Moreover, we analyzed the expression of senescence markers in mouse heart tissues via western blotting. Western blot analysis revealed significant downregulation of SIRT3 expression (Fig. 4C and D) and significant upregulation of p53, p21, and p16 expression in the DCM group (Fig. 4D and E). Pretreatment with CVB-D alleviated the damage induced by DCM. These results showed that CVB-D suppressed the upregulation of these senescence markers.

Next, we investigated whether CVB-D had a similar effect on cells. First, SIRT3 activity measurements revealed that treatment with CVB-D significantly reversed the decrease in SIRT3 activity in HG/PA-induced NMVMs (Fig. 4F). Moreover, SIRT3 protein levels were downregulated in HG/PA-induced NMVMs (Fig. 4G and H), accompanied by upregulation of p53, p21, and p16 (Fig. 4G and I), and these changes were reversed by treatment with CVB-D. Furthermore, the effect of CVB-D on the cell cycle was evaluated in HL-1 cells to assess cell senescence. Flow cytometry analysis revealed that the majority of cells in the HG/PA-treated group were arrested in the G0/G1 phase, with a decrease in the proportion of cells in the S or G2 phase, and this effect was reversed by CVB-D (Fig. 4J and K).

### SIRT3 plays an indispensable role in CVB-D-mediated protection against mitochondrial dysfunction

**Fig. 5:**
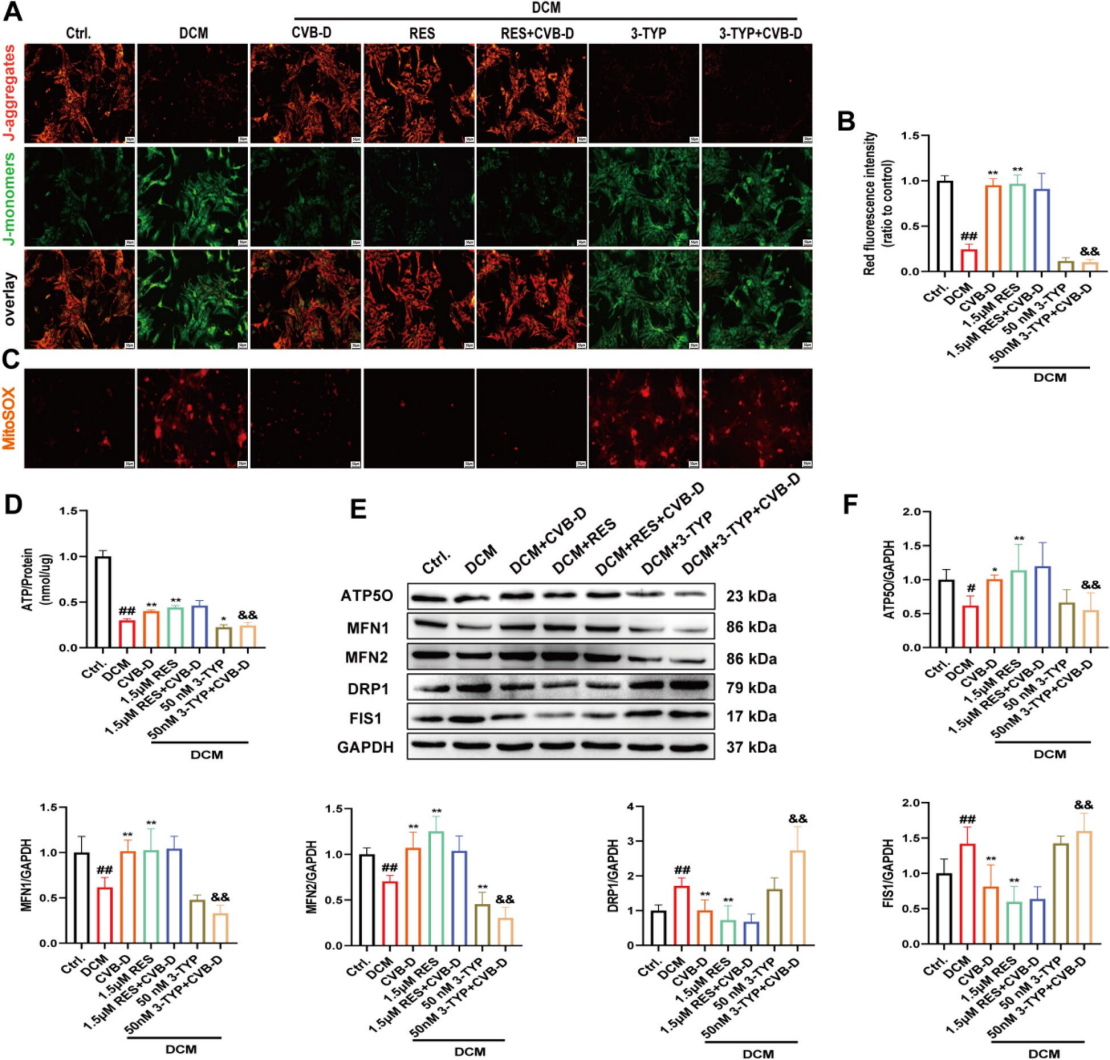
CVB-D ameliorates HG/PA-induced mitochondrial dysfunction by upregulating SIRT3. **A** and **B** The impact of RES or 3-TYP on the ΔΨm of NMVMs (n = 5, scale bar = 50 µm). **C** The impact of RES or 3-TYP on the MitoSOX fluorescence of NMVMs (scale bar = 50 µm). **D** ATP content of NMVMs (n = 3). **E** and **F** The expression levels of ATP5O, MFN1, MFN2, DRP1, and FIS1 after RES or 3-TYP pretreatment (n = 5). ^*#*^*p* < 0.05, ^*##*^*p* < 0.01 versus the control group; ^*^p < 0.05, ^**^p < 0.01 versus the model group; ^*&*^*p* < 0.05, ^*&&*^*p* < 0.01 versus the CVB-D group

To elucidate the role of SIRT3 in the protective effects of CVB-D against HG/PA-induced mitochondrial dysfunction, we used the SIRT3 agonist resveratrol (RES, 1.5 μmol/L) and the inhibitor 3-TYP (50 nmol/L) to determine whether SIRT3 is a crucial mediator of CVB-D protection. After CVB-D preconditioning of NMVMs in vitro, we measured the ΔΨm and other mitochondrial function markers. We found that RES increased ΔΨm and ATP levels (Fig. 5A, B and D), decreased mitochondrial ROS levels (Fig. 5C), and upregulated the expression of mitochondrial function-related proteins such as ATP5O, MFN1, and MFN2 (Fig. 5E and F). The expression of mitochondrial function-related proteins DRP1 and FIS1 was also downregulated (Fig. 5E and F). There were no significant differences observed in NMVMs preconditioned with a combination of CVB-D and RES compared with those preconditioned with RES alone. In contrast, 3-TYP treatment decreased ΔΨm and ATP levels (Fig. 5A, B and D), increased ROS levels (Fig. 5C), upregulated the expression of mitochondrial fission-related proteins DRP1 and FIS1 (Fig. 5E and F) and downregulated the expression of mitochondrial function-related proteins ATP5O, MFN1, and MFN2 (Fig. 5E and F). 3-TYP abolished the protective effects of CVB-D against HG/PA-induced mitochondrial dysfunction.

**Fig. 6:**
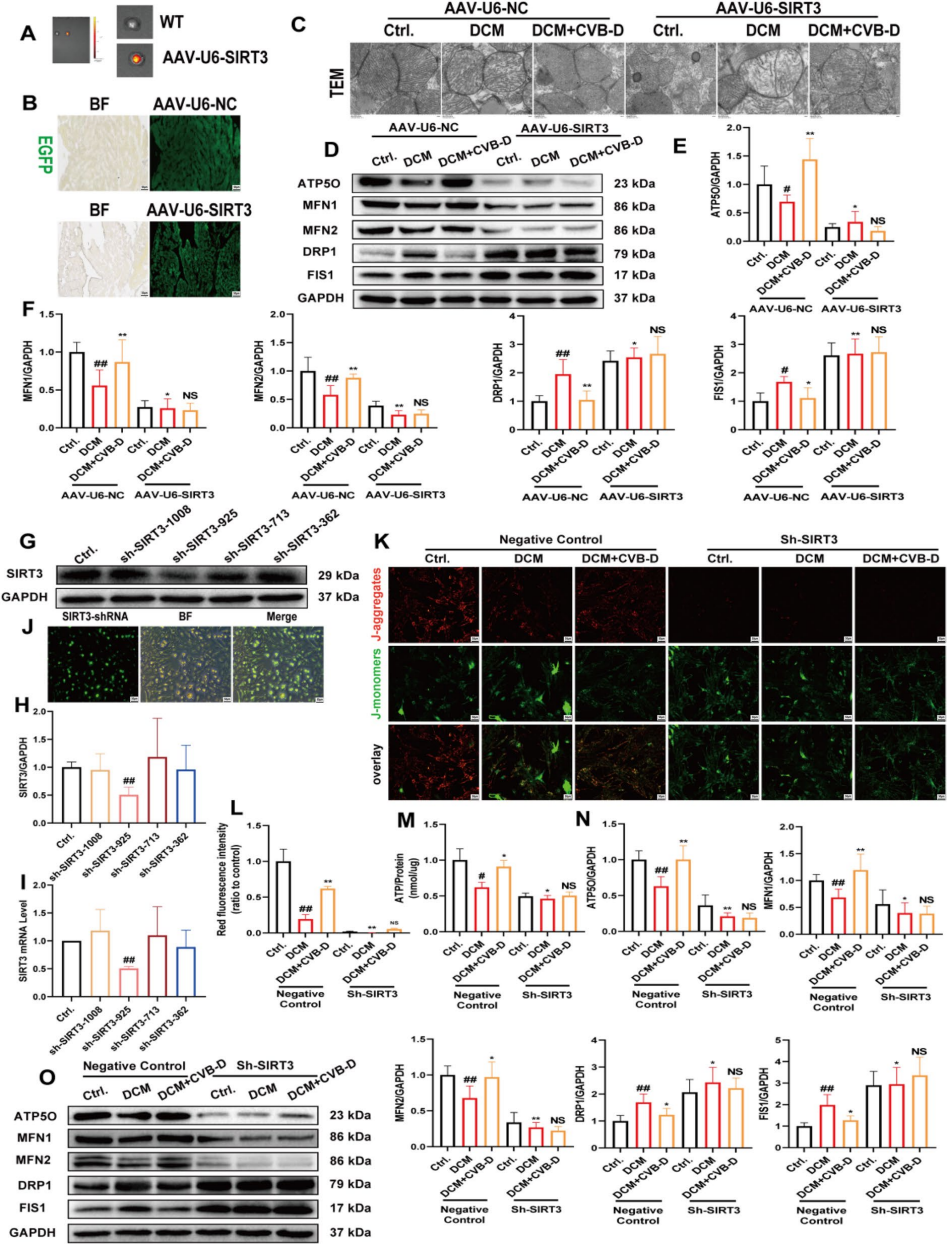
Knockdown of SIRT3 reverses CVB-D-mediated mitochondrial dysfunction inhibition in vivo and in vitro. **A** Representative ex vivo bioluminescence images of heart from the SIRT3-knockdown and control groups. **B** Images showing EGFP fluorescence in the heart infected with AAV-U6-EGFP (scale bar = 50 µm). **C** Representative TEM images of mouse hearts after AAV-U6-EGFP injection (scale bar = 500 nm). **D**–**F** Western blotting analysis of ATP5O, MFN1, MFN2, DRP1, and FIS1 proteins in AAV-U6-EGFP-transfected mice (n = 5). **G** and **H** Western blotting for SIRT3 proteins in NMVMs transfected with negative control or SIRT3 shRNA (n = 3). **I** qRT‒PCR analysis of SIRT3 mRNA levels in NMVMs transfected with negative control or SIRT3 shRNA (n = 3). **J** Transfection efficiency of SIRT3 shRNA (scale bar = 50 µm). **K** and **L** The impact of SIRT3 shRNA on the ΔΨm of NMVMs (n = 5, scale bar = 50 µm). **M** The impact of SIRT3 shRNA on the ATP content of NMVMs (n = 3). **N** and **O** The expression levels of ATP5O, MFN1, MFN2, DRP1, and FIS1 after SIRT3 shRNA treatment (n = 5). ^*#*^*p* < 0.05, ^*##*^*p* < 0.01 versus the control group; ^*^p < 0.05, ^**^p < 0.01 versus the model group

To confirm the essential role of SIRT3 in mitochondrial function in vivo, we established a mouse model in which SIRT3 was specifically knocked down in cardiac cells. This was achieved by intravenous injection of adeno-associated virus 9 vectors expressing shRNA targeting SIRT3 (AAV9-U6-SIRT3) (5′-CTGAGTCCTCGAAGGAAAGAT-3′) and its negative control (AAV9-U6-NC) (5′-ACTACCGTTGTTATAGGTG-3′) into mice for 4 weeks. Ex vivo heart imaging data revealed specific knockdown of SIRT3 in the cardiac tissue (Fig. 6A). Additionally, fluorescence microscopy results demonstrated the expression of exogenous enhanced green fluorescent protein (EGFP) in mouse hearts (Fig. 6B). Additionally, we used TEM to examine alterations in mitochondrial ultrastructure. Reduced SIRT3 expression was associated with marked mitochondrial fragmentation and abnormal crista morphology. Treatment with AAV9-U6-SIRT3 blocked the mitochondrial protective effects of CVB-D (Fig. 6C). Mitochondrial dynamics are regulated by MFN1, MFN2, DRP1, and FIS1 proteins. Treatment with AAV9-U6-SIRT3 was associated with decreased expression of mitochondrial function-related proteins ATP5O, MFN1, and MFN2 and increased expression of mitochondrial fission-related proteins DRP1 and FIS1 (Fig. 6D, E and F). Moreover, the IF results were consistent with the Western blot results (**Fig. S5**).

Simultaneously, we investigated the effects of SIRT3 deficiency on mitochondrial function in NMVMs. The reduction in SIRT3 levels was confirmed by qRT‒PCR analysis (Fig. 6I), western blot analysis (Fig. 6G and H) and IF staining (Fig. 6J), indicating that sh-SIRT3-925-knockdown cells were suitable for further experiments. We measured the ΔΨm and ATP levels in NMVMs and found that sh-SIRT3 reduced both ΔΨm and ATP levels (Fig. 6K, L and M). As expected, we evaluated the effect of sh-SIRT3 on mitochondrial function in NMVMs via western blot analysis. The results in NMVMs were consistent with those from the in vivo studies (Fig. 6N and O).

### SIRT3 plays an indispensable role in CVB-D-mediated protection against senescence

**Fig. 7:**
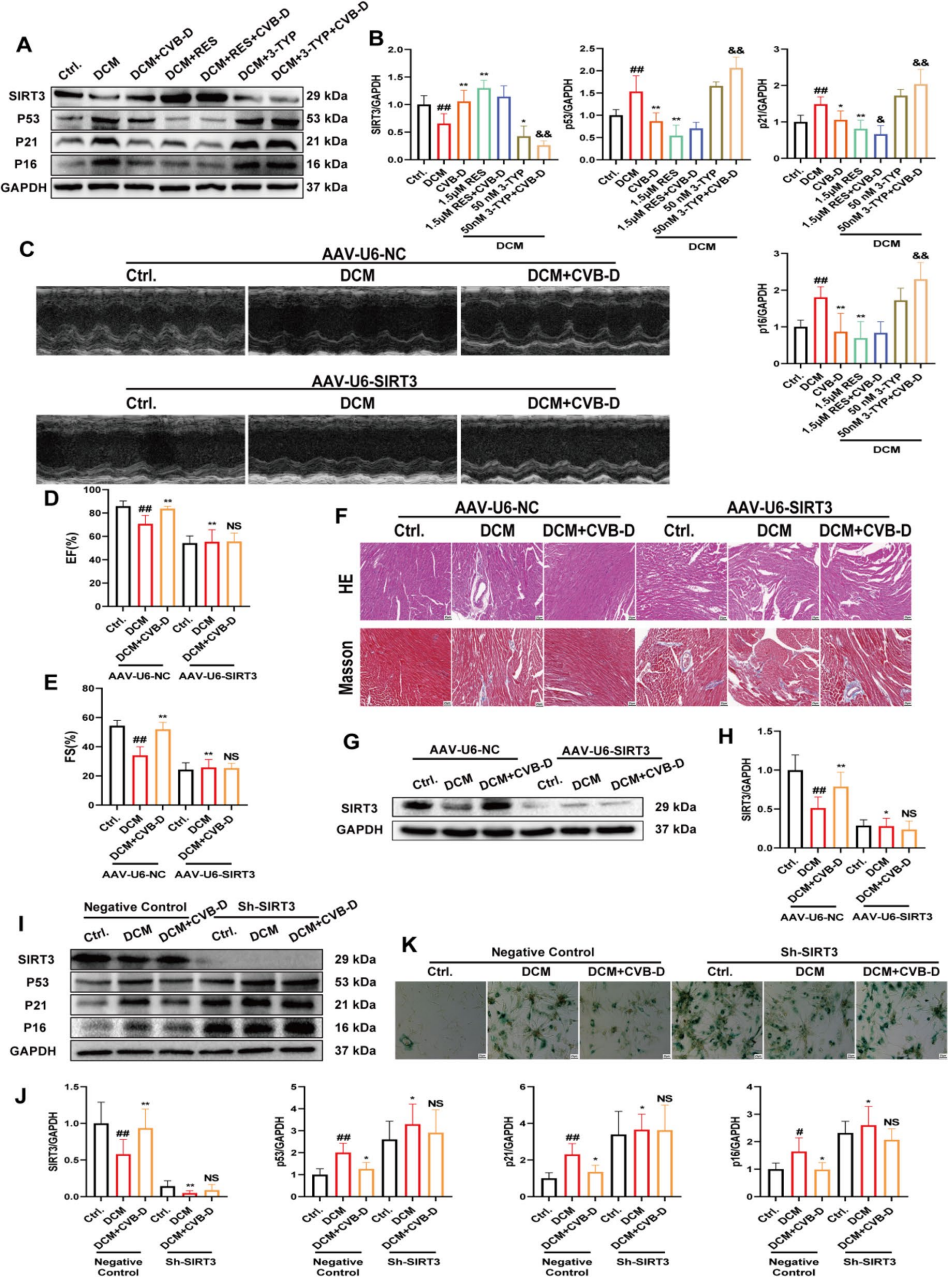
SIRT3 plays an indispensable role in CVB-D-mediated protection against senescence. **A** and **B** The expression levels of SIRT3, p53, p21, and p16 after RES or 3-TYP pretreatment in NMVMs (n = 5). **C** Representative M-mode echocardiographic images. **D** and **E** Quantitative analysis of data for the EF% and FS% (n = 6). **F** Heart tissue samples were subjected to H&E (HE) and Masson’s trichrome staining (scale bar = 25 µm). **G** and **H** Western blot analysis of SIRT3 protein in the hearts of the AAV-U6-EGFP-transfected mice (n = 5). **I** and **J** The expression levels of SIRT3, p53, p21, and p16 after SIRT3 shRNA treatment in NMVMs (n = 5). **K** SA-GAL activity was determined via β-gal staining (scale bar = 25 µm). ^*#*^*p* < 0.05, ^*##*^*p* < 0.01 versus the control group; ^*^p < 0.05, ^**^p < 0.01 versus the model group; ^*&*^*p* < 0.05, ^*&&*^*p* < 0.01 versus the CVB-D group

We investigated whether SIRT3 has a similar role in senescence. We assessed the effects of the SIRT3 agonist RES and the inhibitor 3-TYP on the expression of senescence-related proteins via western blotting. The results showed that the RES agonist inhibited the expression of senescence-related proteins and promoted SIRT3 expression. Conversely, 3-TYP increased the expression of senescence-related proteins, decreased SIRT3 expression, and reversed the protective effect of CVB-D (Fig. 7A and B). To further explore the role of SIRT3, we generated SIRT3 knockout models in animals and SIRT3 knockdown models in cells. Echocardiography revealed that CVB-D improved cardiac function; however, this protective effect was abolished when SIRT3 was knocked down (Fig. 7C, D and E).

Additionally, serum levels of LDL-C, TG, and TC were significantly higher, while HDL-C levels were significantly lower in the DCM group than in the control group. CVB-D effectively reversed these lipid abnormalities. However, SIRT3 knockdown abolished the beneficial effect of CVB-D (**Fig. S6**). H&E and Masson's trichrome staining results showed that AAV-U6-SIRT3 transfection exacerbated heart damage and blocked the therapeutic effects of CVB-D (Fig. 7F). IF analysis was used to detect the expression levels of senescence markers. The results showed that administration of AAV-U6-SIRT3 upregulated the expression of senescence markers (p53, p21, and p16) and impaired the protective effect of CVB-D (**Fig. S7**). As expected, western blotting results showed that SIRT3 knockdown further decreased SIRT3 protein expression (Fig. 7G and H). Furthermore, in vitro assays of SIRT3 and senescence-related protein expression, along with western blotting, showed that compared with the DCM group, the CVB-D treatment group presented decreased expression of senescence-related proteins and increased expression of SIRT3. However, only when SIRT3 expression was further reduced after SIRT3 knockdown did the expression of senescence-related proteins increase significantly, and CVB-D lost its protective effect (Fig. 7I and J). Meanwhile, we performed β-gal staining to label senescent NMVMs. The β-gal staining were consistent with those of western blotting (Fig. 7K).

### The protective impact of CVB-D depends on the deacetylation of ATP5O by SIRT3

**Fig. 8:**
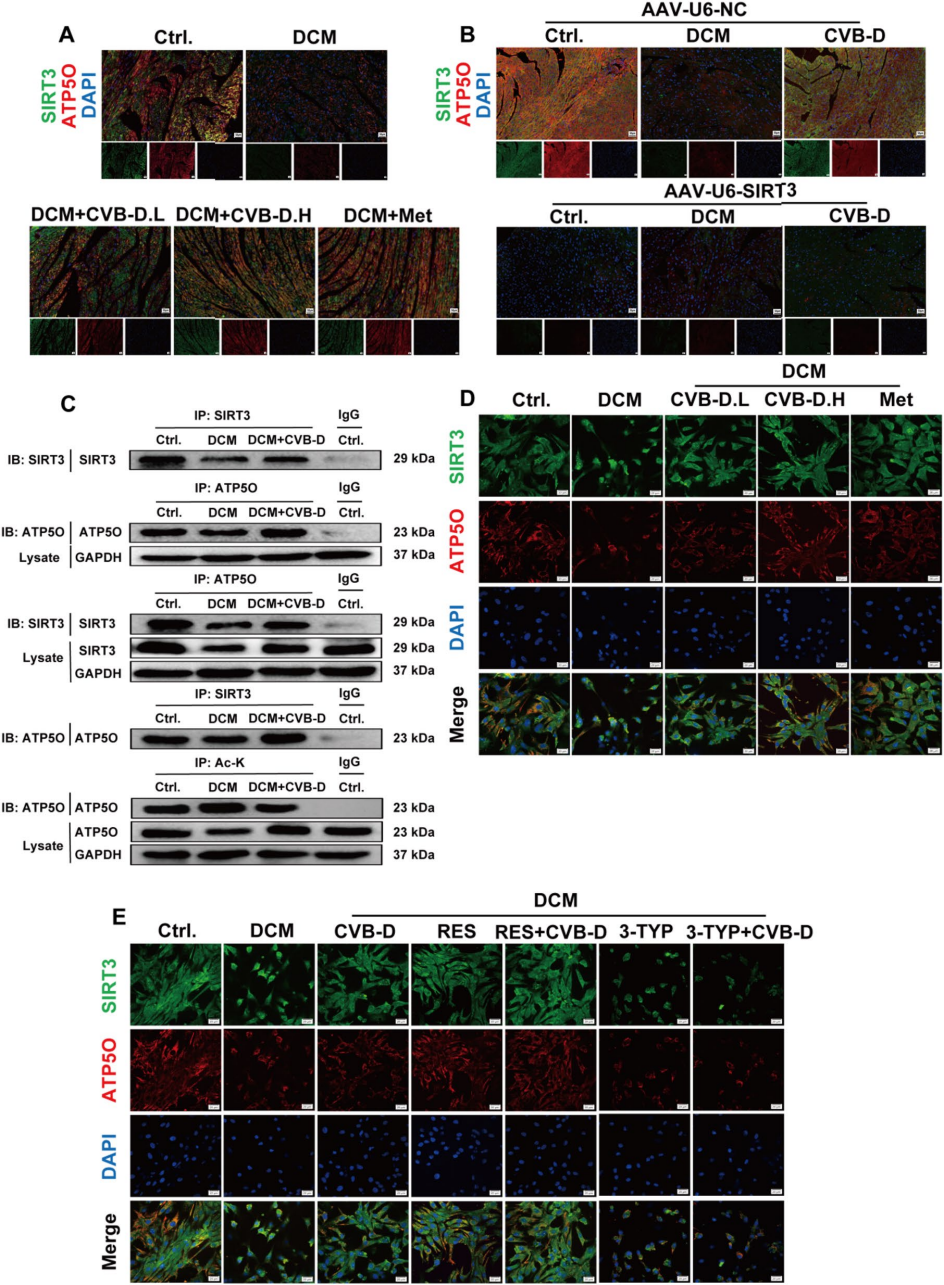

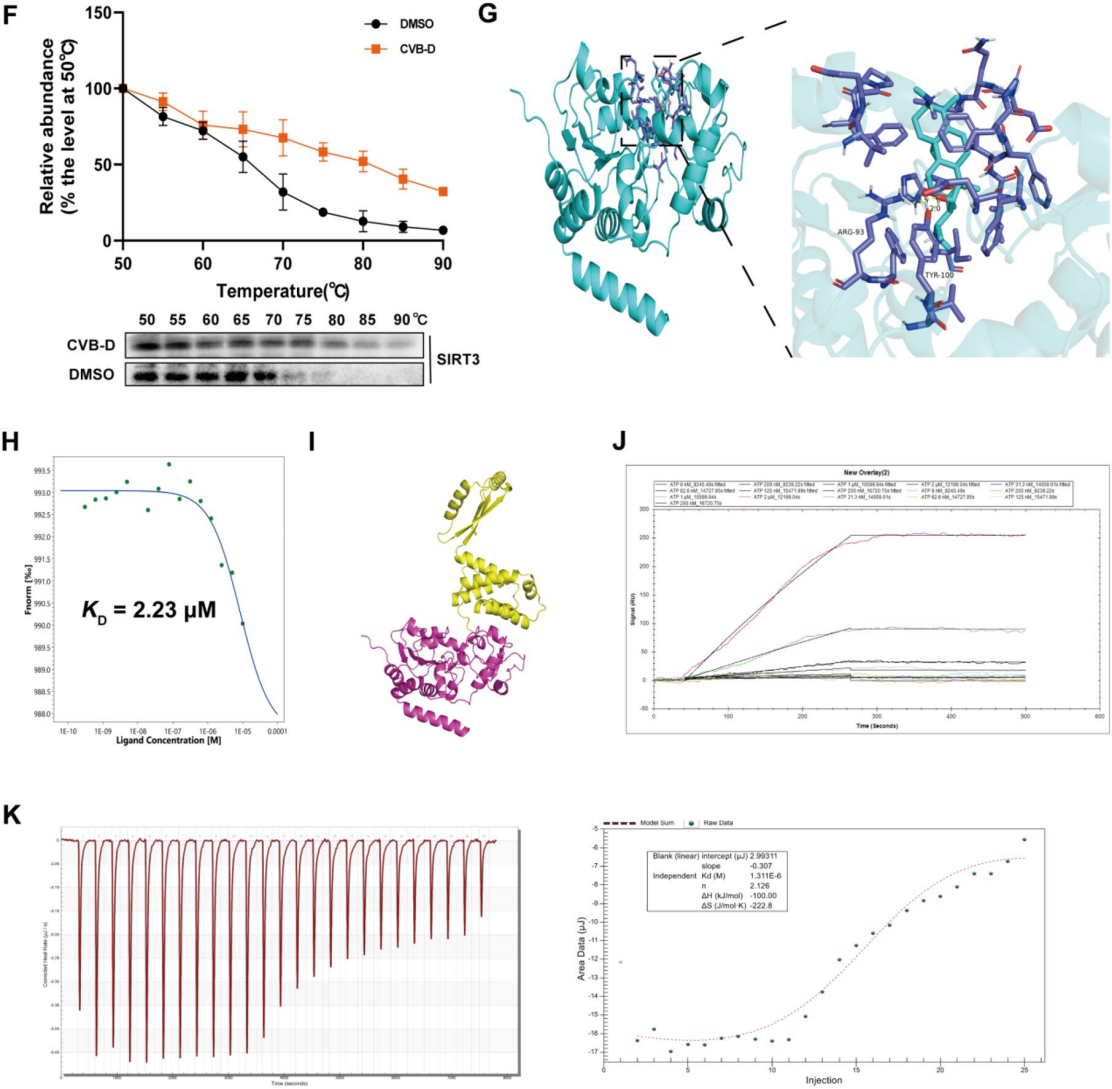
The protective effect of CVB-D depends on the deacetylation of ATP5O by SIRT3. **A** and **B** IF images of SIRT3 and ATP5O were colocalised in the myocardial tissue (scale bar = 50 or 20 µm). **C** Immunoprecipitation analysis revealed that pretreatment with CVB-D inhibited ATP5O acetylation in NMVMs (n = 3). **D** and **E** IF images of SIRT3 and ATP5O colocalization in NMVMs (scale bar = 20 µm). **F** CETSA was performed on NMVMs treated with CVB-D or DMSO for 48 h (n = 3). **G** Molecular docking of CVB-D to SIRT3 protein. **H** MST analysis of the CVB-D and SIRT3 interactions. **I** Molecular docking between SIRT3 and ATP5O protein. **J** Binding affinity analysis of SIRT3 and ATP5O determined by SPR. **K** Isothermal titration calorimetry (ITC) assay exhibited that SIRT3 exhibited a binding affinity for ATP5O

The relationship between SIRT3 and ATP5O in mitochondria plays a crucial role in the protective effects of CVB-D. We conducted an immunofluorescence colocalization assay and found that SIRT3 and ATP5O were colocalized in myocardial tissue. Compared with the control group, the levels of SIRT3 and ATP5O colocalization were lower in the DCM group, while CVB-D pretreatment restored these levels (Fig. 8A). Interestingly, we established a mouse model in which SIRT3 was specifically knocked down in cardiac cells via intravenous injection of AAV9-U6-SIRT3. This led to the loss of the protective effects of CVB-D and further reductions in SIRT3 and ATP5O expression (Fig. 8B). To further explore the interaction between SIRT3 and ATP5O, we investigated their relationship in NMVMs. Immunoprecipitation (IP) experiments showed that the association between SIRT3 and ATP5O was weaker in the DCM group than in the control group. Moreover, SIRT3 has the capacity to deacetylate ATP5O. The level of ATP5O acetylation was increased in the DCM group, which was reversed by CVB-D treatment, leading to a decrease in ATP5O acetylation (Fig. 8C). We also observed the colocalization of SIRT3 and ATP5O in NMVMs via IF staining, which was consistent with the results of the animal experiments (Fig. 8D). Notably, the mere colocalization of SIRT3 and ATP5O is insufficient. Therefore, we used the SIRT3 agonist RES and the inhibitor 3-TYP for subsequent interference studies. The effects of the SIRT3 agonist RES and the inhibitor 3-TYP on the colocalization of SIRT3 and ATP5O were investigated using IF assay. The results showed that RES promoted the colocalization of SIRT3 and ATP5O and reversed HG/PA-induced damage, whereas 3-TYP inhibited their colocalization and counteracted the protective effects of CVB-D (Fig. 8E). We performed a cellular thermal shift assay (CETSA) to determine whether CVB-D interacts with SIRT3. The results showed that CVB-D significantly increased the thermal stability of SIRT3 at different temperatures (Fig. 8F). Molecular docking analysis between the CVB-D molecule and SIRT3 protein revealed an energy release of -261.7 kcal/mol (Fig. 8G). Moreover, microscale thermophoresis (MST) analysis showed a specific interaction between CVB-D and SIRT3, with a dissociation constant (KD) of 2.23 μM (Fig. 8H). Meanwhile, molecular docking analysis of the SIRT3 protein and the ATP5O protein revealed an energy release of -674.9 kcal/mol (Fig. 8I). To further elucidate the relationship between SIRT3 and ATP5O proteins, we used a surface plasmon resonance (SPR)-based competition assay to examine the direct binding between them. The results showed that SIRT3 and ATP5O proteins bind to each other with high affinity, with an association rate constant (ka) of 8.50 × 10^2^ (1/(M·s)), a dissociation rate constant (kd) of 5.54 × 10⁻⁸ (1/s), and a dissociation constant (KD) of 6.53 × 10⁻^11^ (M) (Fig. 8J). To comprehensively elucidate the relationship between SIRT3 and ATP5O, we conducted isothermal titration calorimetry (ITC) experiments. The results showed that SIRT3 directly bound to ATP5O with a dissociation constant (KD) of 1.31 μM (Fig. 8K). Collectively, these findings suggest that CVB-D activates SIRT3, thereby promoting its deacetylation function. In turn, SIRT3 increases the level of deacetylated ATP5O, which may contribute to the alleviation of DCM progression.

### SIRT3 deacetylates ATP5O at the K162 site

**Fig. 9:**
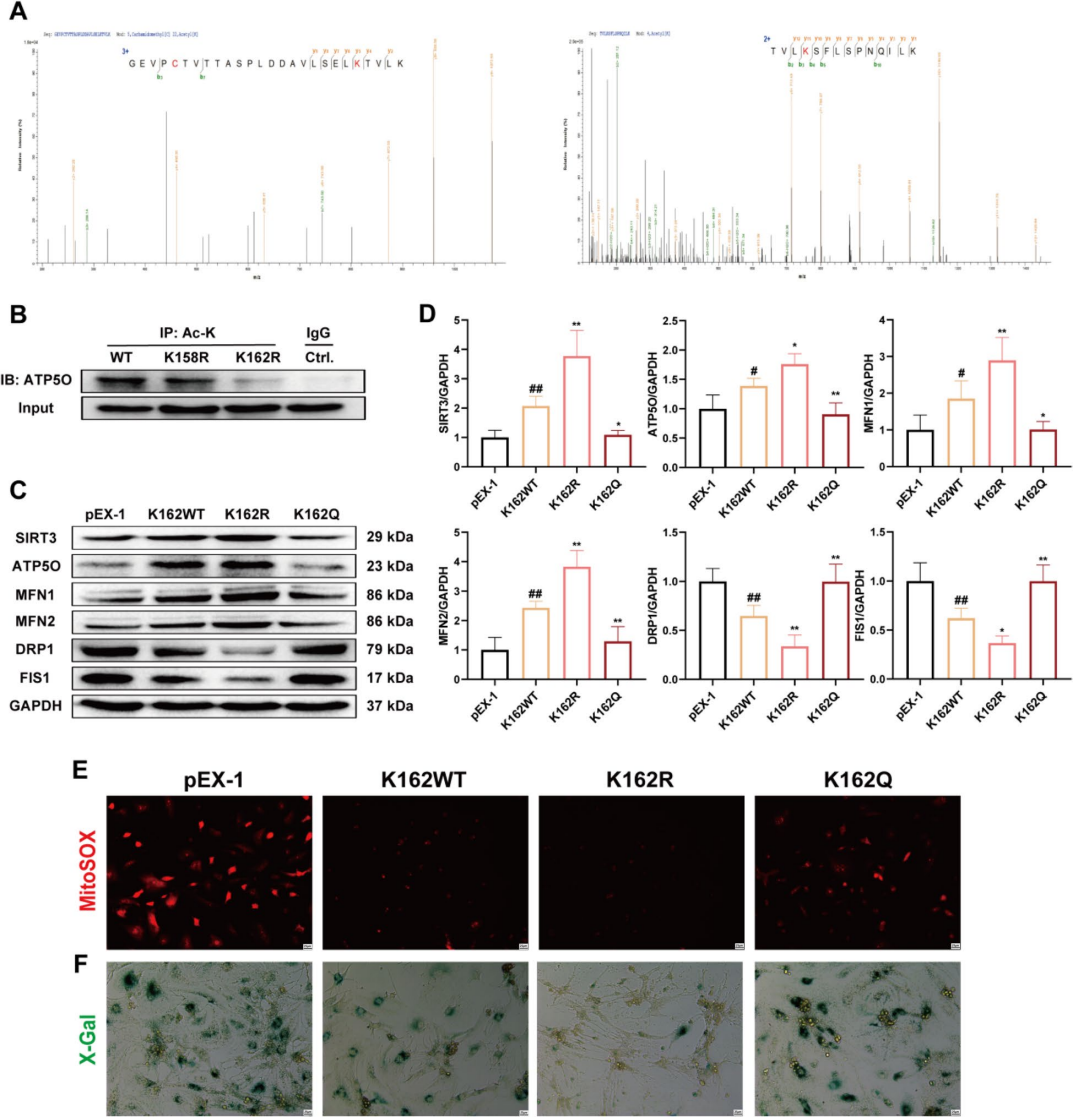
SIRT3 deacetylates ATP5O at the K162 site. **A** LC‒MS/MS was used to analyze the acetylation sites of ATP5O. **B** Immunoprecipitation analysis of mutations at ATP5O acetylation sites in NMVMs (n = 3). **C** and **D** Western blotting analysis expression levels of SIRT3, ATP5O, MFN1, MFN2, DRP1 and FIS1 in NMVMs (n = 5). **E** The impact of mutations at ATP5O acetylation sites on the MitoSOX fluorescence of NMVMs (scale bar = 25 µm). **F** SA-GAL activity was determined via β-gal staining (scale bar = 25 µm). ^*#*^*p* < 0.05, ^*##*^*p* < 0.01 versus the pEX-1 group; ^*^p < 0.05, ^**^p < 0.01 versus the K162WT group

As demonstrated in previous experiments, the protective effect of CVB-D relies on SIRT3-mediated deacetylation of ATP5O. However, the specific location of this deacetylation event remains unclear. Thus, we identified the potential acetylation sites of ATP5O, namely K158 and K162, via LC‒MS/MS analysis (Fig. 9A). We mutated the lysine residues at positions K158 and K162 to arginines to mimic the deacetylated state and then separately transfected NMVMs with these mutant constructs. IP results clearly showed that transfection with the K162 mutant alone reduced the acetylation level of ATP5O in NMVMs, whereas the acetylation level of ATP5O did not significantly change in NMVMs transfected with the K158 mutant. These results indicated that the site on ATP5O deacetylated by SIRT3 is K162 (Fig. 9B). To further validate the effect of mutations at the ATP5O acetylation site, we synthesized plasmids harboring the deacetylated K162R mutation and the acetylated K162Q mutation. The western blotting results revealed that, in the ATP5O-K162R group, the expression levels of SIRT3, ATP5O, MFN1, and MFN2 were increased, while the expression of DRP1 and FIS1 were decreased. In contrast, compared with the ATP5O-WT group, the ATP5O-K162Q group presented decreased expression of SIRT3, ATP5O, MFN1, and MFN2 proteins and increased expression of DRP1 and FIS1 proteins (Fig. 9C and D). Additionally, we conducted MitoSOX fluorescence and β-gal staining experiments, and the results were consistent with those of the western blotting analysis (Fig. 9E and F).

### The protective effect of SIRT3 on DCM partially depends on ATP5O

**Fig. 10:**
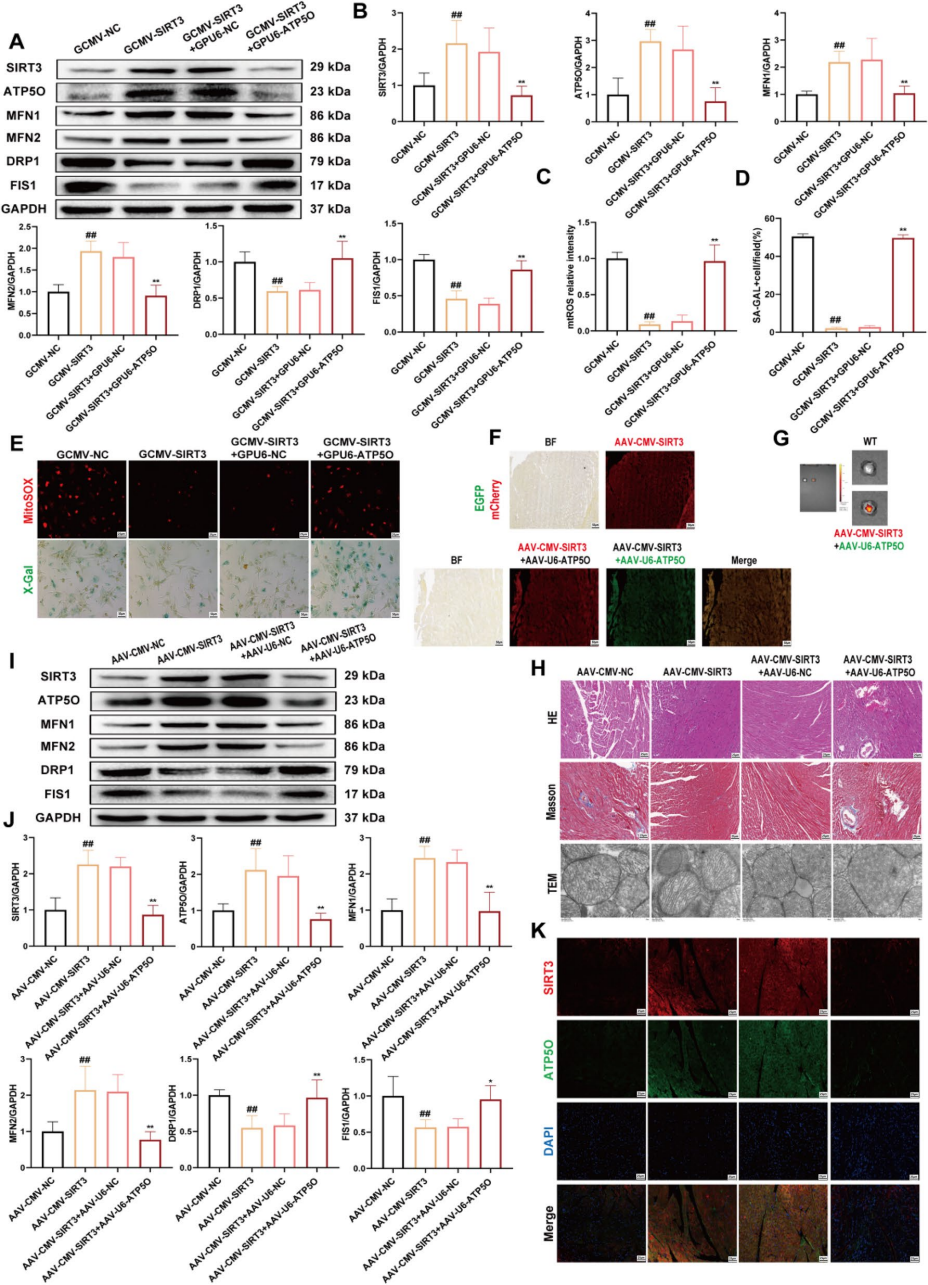
The protective effect of SIRT3 on DCM partially depends on ATP5O. **A** and **B** Western blotting analysis expression levels of SIRT3, ATP5O, MFN1, MFN2, DRP1 and FIS1 in NMVMs (n = 5). **C** and **E** Quantification of MitoSOX fluorescence in NMVMs via ImageJ (n = 5, scale bar = 50 µm). **D** and **E** SA-GAL activity was determined via β-gal staining (n = 5, scale bar = 50 µm). ^*#*^*p* < 0.05, ^*##*^*p* < 0.01 versus the GCMV-NC group; ^*^p < 0.05, ^**^p < 0.01 versus the GCMV-SIRT3 group. **F** Images showing EGFP and mCherry fluorescence in the hearts infected with AAV9-CMV-SIRT3 and AAV9-U6-ATP5O (scale bar = 50 µm). **G** Representative ex vivo bioluminescence images of hearts from mice in the AAV9-CMV-SIRT3, AAV9-U6-ATP5O and control groups. **H** Heart tissue samples were dyed with H&E (HE) and Masson’s trichrome (scale bar = 25 µm), and transmission electron microscopy (TEM) images of mitochondria (scale bar = 50 nm). **I** and **J** Western blot analysis expression levels of SIRT3, ATP5O, MFN1, MFN2, DRP1 and FIS1 in myocardial tissue (n = 5). **K** IF images of SIRT3 and ATP5O were colocalization in myocardial tissue (scale bar = 20 µm). ^*#*^*p* < 0.05, ^*##*^*p* < 0.01 versus the AAV-CMV-NC group; ^*^p < 0.05, ^**^p < 0.01 versus the AAV-CMV-SIRT3 group

We investigated whether ATP5O is involved in dilated DCM via SIRT3-mediated deacetylation. To this end, we transfected NMVMs with a plasmid for ATP5O knockdown (GPU6-ATP5O) (5′-CGGCCAACCTCATGAATTTAC-3′) and a plasmid for SIRT3 overexpression (GCMV-SIRT3) (NM_001127351.1) prior to HG/PA treatment. In HG/PA-treated NMVMs, compared with the SIRT3 blank vector plasmid group (GCMV-NC), the protein expression levels of SIRT3, ATP5O, MFN1, and MFN2 were upregulated in the GCMV-SIRT3 group, whereas the expression levels of DRP1 and FIS1 were downregulated (Fig. 10A and B). Moreover, MitoSOX fluorescence staining analysis showed that the fluorescence intensity of GCMV-SIRT3 group was decreased, indicating that the ROS content of mitochondria decreased (Fig. 10C and E). Additionally, β-gal staining results demonstrated significant inhibition of NMVM senescence in the GCMV-SIRT3 group (Fig. 10D and E). However, simultaneous knockdown of ATP5O largely abolished the beneficial effects of SIRT3 overexpression.

We aimed to determine whether the SIRT3‒ATP5O axis could also delay cardiomyocyte senescence by regulating mitochondrial function in animal models. We established a mouse model in which SIRT3 was overexpressed and ATP5O was simultaneously knocked down specifically in cardiac cells. This was achieved by intravenous injection of adeno-associated virus 9 vectors AAV9-CMV-SIRT3, AAV9-U6-ATP5O, and their negative controls (AAV9-CMV-NC and AAV9-U6-NC) into mice for 4 weeks. We first assessed the cardiac function of mice using echocardiography. The results showed that SIRT3 overexpression improved cardiac function parameters in mice, whereas ATP5O knockdown reversed these protective effects (**Fig. S8**). Moreover, SIRT3 overexpression increased the serum levels of HDL-C and decreased the levels of LDL-C and TGs. However, ATP5O knockdown reversed the protective effects of SIRT3 overexpression. Notably, neither SIRT3 overexpression nor ATP5O knockdown affected the serum TC content (**Fig. S9**). The fluorescence microscopy results revealed the expression of exogenous enhanced green fluorescent protein (EGFP) and mCherry protein in the mouse heart (Fig. 10F). Furthermore, ex vivo heart imaging data revealed that SIRT3 overexpression and ATP5O knockdown were successfully achieved in cardiac tissue (Fig. 10G). Histopathological analysis (H&E and Masson's staining) showed that, compared with the AAV-CMV-NC group, the AAV-CMV-SIRT3 group presented an organized myocardial fiber arrangement, normal cardiomyocyte nuclei, and significantly reduced collagen content. However, the beneficial effects of AAV-CMV-SIRT3 on myocardial histopathological morphology were abolished by AAV-U6-ATP5O (Fig. 10H). We used TEM to examine changes in mitochondrial ultrastructure. Compared with the AAV-CMV-NC group, mitochondria in the AAV-CMV-SIRT3 group exhibited moderate fragmentation and normal crista morphology. However, treatment with AAV-U6-ATP5O blocked the protective effects of AAV-CMV-SIRT3 on mitochondria (Fig. 10H). Western blot analysis revealed that the expression of mitochondrial dynamics-related proteins MFN1 and MFN2 was increased, whereas the expression of DRP1 and FIS1 proteins was decreased in the AAV-CMV-SIRT3 group compared with the AAV-CMV-NC group. However, co-treatment with AAV-U6-ATP5O and AAV-CMV-SIRT3 impaired mitochondrial function and abolished the protective effects of AAV-CMV-SIRT3 (Fig. 10I and J). IF detection of mitochondrial dynamics-related and senescence-related proteins showed that, compared with the AAV-CMV-NC group, the expression levels of MFN1 and MFN2 were increased, whereas the protein levels of DRP1, FIS1, p53, p21, and p16 were decreased in the AAV-CMV-SIRT3 group (**Fig. S10**). Notably, western blot and immunofluorescence colocalization analyses revealed that, compared with the AAV-CMV-NC group, the AAV-CMV-SIRT3 group presented increased SIRT3 and ATP5O protein levels and increased fluorescence colocalization. However, ATP5O knockdown led to a decrease in both SIRT3 and ATP5O protein levels and fluorescence colocalization (Fig. 10I, J and K). In conclusion, our findings indicate that SIRT3 exerts a protective effect against aging induced by mitochondrial dysfunction through the regulation of ATP5O.

## Discussion

In 1972, Shirley Rubler discovered DCM, indicating that this typical myocardial disease is caused by cardiac hypertrophy and diabetic microangiopathy and has an extremely high lethality rate worldwide [23]. CVB-D is a triterpenoid alkaloid derived from Buxus microphylla and has been used to treat cardiovascular diseases, such as cardiac arrhythmias and coronary heart disease [24]. Nevertheless, the therapeutic target molecules of CVB-D for DCM remain unclear, which limits its pharmacological research and clinical application. To determine whether CVB-D could improve DCM, we conducted the experimental studies described above. Our results showed that CVB-D alleviated DCM-induced mitochondrial dysfunction, thereby delaying cardiomyocyte senescence both in vivo and in vitro. In this study, we identified SIRT3 as a direct binding target of ATP5O. Additionally, we found that CVB-D promoted SIRT3 expression, which was associated with ATP5O deacetylation and improved mitochondrial function (Fig. 8). These results provide new insights into the specific targets of the protective effect of CVB-D and its pharmacological mechanisms in DCM.

The heart is the organ with the highest oxygen consumption in the body and requires a large amount of energy to maintain its function [25]. Mitochondria produce ATP, which is regarded as the primary energy source in cardiomyocytes and is essential for maintaining the body's normal functions [26, 27]. Mitochondrial dysfunction is often accompanied by reduced ATP synthesis and decreased respiratory capacity, and it is also an initial factor in myocardial infarction and cellular senescence [28, 29]. We used TEM to observe the morphology of mitochondria in mice to determine whether the improvement in DCM caused by CVB-D is related to mitochondrial protection. The DCM group exhibited mitochondrial crista disruption, severe mitochondrial swelling, and mitochondrial rupture. However, treatment with CVB-D significantly improved the morphology of mitochondria. There is no doubt that ATP synthesis and ATP synthase activity were reduced in the DCM group, and this trend could be reversed by pretreatment with CVB-D (Fig. 2). We measured ATP synthesis and ATP synthase activity levels. The results were consistent with those of animal experiments (Fig. 3). These results indicate that the ameliorative effect of CVB-D on DCM may be associated with mitochondrial protection.

The relevant literature indicates that the fission–fusion process of mitochondria is crucial for heart function and that mitochondrial dysfunction can accelerate the manifestation of the aging phenotype [30, 31]. Our study revealed that in the DCM group, there was increased expression of senescence-related proteins and positive β-gal staining. Moreover, the majority of HL-1 cells were arrested in the G0/G1 phase. However, treatment with CVB-D alleviated the progression of senescence (Fig. 4). These data support the hypothesis that CVB-D can protect cardiomyocytes from senescence by improving mitochondrial function. However, the specific mechanism by which CVB-D enhances mitochondrial function remains to be fully elucidated and warrants further investigation.

We investigated the sirtuin protein family, particularly SIRT3, which possesses deacetylase activity and is associated with human longevity [32, 33], to elucidate the mechanism by which CVB-D protects against mitochondrial dysfunction and delays cardiomyocyte senescence. SIRT3 is regarded as the major mitochondrial deacetylase [34, 35]. The results of our experiments using the SIRT3 agonist RES and the inhibitor 3-TYP in NMVMs were intriguing. These results showed that SIRT3 is essential for the protective effects of CVB-D against both mitochondrial dysfunction and cardiomyocyte senescence (Figs. 5 and 6). The results of our in vivo animal experiments and in vitro NMVM experiments using AAV9-U6-SIRT3 and sh-SIRT3 vectors revealed that the protective effects of CVB-D against DCM are dependent on SIRT3 expression (Fig. 7). However, the protective effects of SIRT3 downstream targets against DCM remain unknown. Hence, further studies are needed to explore the downstream targets of SIRT3.

Next, we will focus on ATP5O, a downstream target molecule of SIRT3. The ATP5O protein is an important differentially expressed protein in mitochondria, which is involved in efficient ATP production and plays a crucial role in maintaining the structural stability of F1F0 ATP synthase [36]. Related studies have shown that ATP5O protein levels significantly influence mitochondrial function and the aging process [37]. Our in vitro and in vivo results revealed a significant decrease in ATP5O protein levels in the DCM group, followed by a significant increase after pretreatment with CVB-D (Figs. 2 and 3).

Immunofluorescence colocalization and IP experiments revealed an interaction between SIRT3 and ATP5O, and CVB-D significantly affected the strength of this interaction. Molecular interaction assays directly demonstrated the binding between SIRT3 and ATP5O, and CVB-D promoted this binding (Fig. 8). However, the question remains: what occurs when SIRT3 binds to ATP5O? Given that SIRT3 is a deacetylase, we hypothesized that SIRT3 might deacetylate ATP5O. Indeed, our findings revealed that the acetylation level of ATP5O was increased in the DCM group and decreased after pretreatment with CVB-D (Fig. 8). LC‒MS/MS analysis identified two acetylation sites in the ATP5O protein. Mutation of the ATP5O-K162 residue significantly affected mitochondrial function and the aging process (Fig. 9). Finally, we overexpressed SIRT3 in combination with ATP5O knockdown both in vitro and in vivo and showed that the induction of AAV-CMV-SIRT3 significantly improved mitochondrial dysfunction and cardiomyocyte senescence. However, ATP5O knockdown abolished the protective effects of SIRT3 overexpression (Fig. 10). Overall, the SIRT3‒ATP5O axis plays a crucial role in the process by which CVB-D ameliorates DCM.

While this study identifies the SIRT3–ATP5O axis as a novel mechanism for CVB-D in countering cardiomyocyte senescence, several limitations should be acknowledged. Firstly, the poor aqueous solubility of CVB-D precluded a direct assessment of its pharmacokinetic profile, including oral bioavailability, serum concentration, and cardiac tissue distribution. Establishing a robust quantitative method for CVB-D in biological matrices is therefore a critical prerequisite for future translational studies. Secondly, our mechanistic exploration, while establishing the SIRT3–ATP5O axis, did not fully delineate the upstream and interactive signaling pathways. Given the documented crosstalk between AMPK and SIRT3[38], a key unresolved question is whether CVB-D primarily activates AMPK to indirectly potentiate SIRT3, or if these pathways operate synergistically to promote mitochondrial biogenesis and functional recovery. Future investigations are warranted to address this and other emergent questions: (1) How does SIRT3, in the context of CVB-D-mediated cardioprotection, regulate other mitochondrial targets, such as complexes I-IV of the electron transport chain? (2) Does CVB-D treatment in our DCM model elicit off-target metabolic effects, such as insulin resistance? (3) What accounts for the absence of a significant alteration in serum total cholesterol levels following SIRT3 overexpression or ATP5O knockdown? (4) Are additional subunits of ATP synthase, apart from ATP5O, involved in the mitochondrial dysfunction associated with DCM? Despite these limitations, our work provides the evidence that CVB-D confers protection against cardiac aging by bolstering mitochondrial function via the SIRT3–ATP5O axis. Resolving the aforementioned questions will be instrumental in fully elucidating the drug's mechanism of action and guiding its clinical development.

## Conclusions

Overall, our study identified CVB-D, a potential antiaging agent, as a compound that can mitigate mitochondrial dysfunction in both NMVMs and DCM model mice. Notably, SIRT3, an essential regulator of mitochondrial function, plays a crucial role in the protective effects of CVB-D against DCM. We identified SIRT3 as the direct target of CVB-D and elucidated the precise mechanism by which CVB-D acts on the SIRT3-ATP5O axis to alleviate DCM pathology, restore mitochondrial morphology, and mitigate senescence in DCM mice. Thus, our study reveals a unique mechanism of mitochondrial protection conferred by CVB-D, which may facilitate the identification of novel candidates for DCM intervention.

## Supplementary Information

Additional file 1.

Additional file 2.

Additional file 3.

## Data Availability

The data in this study are available from the corresponding author upon reasonable request.

## Abbreviations

DCM: Diabetic cardiomyopathy
CVB-D: Cyclovirobuxine D
HFD: High-fat diet
STZ: Streptozotocin
SIRT3: Sirtuins 3
NMVMs: Primary neonatal mouse ventricular myocytes
HG/PA: High glucose/Palmitic acid
qRT-PCR: reverse transcription-quantitative PCR
MST: Microscale thermophoresis
SPR: Surface plasmon resonance
ITC: Isothermal titration calorimetry
T2DM: Type 2 diabetes mellitus
ATP5O: ATP synthase subunit O
OXPHOS: Oxidative phosphorylation
BSA: Bovine serum albumin
BCA: Bicinchoninic acid
cTnT: Cardiomytroponin T
CETSA: Cellular thermal shift assay
DAPI: 4', 6-diamidino-2-phenylindole
SA-β-gal: Senescence-associated β-galactosidase
AUCs: Areas under the curve
OGTTs: Oral glucose tolerance test
EF%: Ejection fraction
FS%: Fractional shortening
H&E: Hematoxylin‒eosin
WGA: Wheat germ agglutinin
TEM: Transmission electron microscopy
IF: Immunofluorescence
ΔΨm: Mitochondrial membrane potential
IP: Immunoprecipitation
DCFH-DA: 2’, 7’-Dichlorofluorescin diacetate
DM: Diabetes mullins
DMSO: Dimethyl sulfoxide
DRP1: Dynamin-related protein
FBG: Fasting blood glucose
FBS: Fetal bovine serum
GAPDH: Glyceraldehyde phosphate dehydrogenase
HOMA-IR: Homeostasis model assessment-Insulin resistance
IPGTT: Intraperitoneal glucose tolerance test
MFN1/2: Mitofusin1/2
MMP: Mitochondrial membrane potential
MTT: 3-(4,5-dimethyl-2- thiazolyl)-2,5-diphenyl-2-H-tetrazolium bromide
OCR: Oxygen consumption rate
PBS: Phosphate-buffered saline
PMSF: Phenylmethyl sulfonyl fluoride
PVDF: Polyvinylidene difluoride
RES: Resveratrol
ROS: Reactive oxygen species
SDS-PAGE: SDS-polyacrylamide gel electrophoresis
SOD: Superoxide dismutase
TBST: Tris buffered saline with Tween-20
3-TYP: 3- (1H-1,2,3-triazol-4-yl) pyridine
