# Supplementary material for: Cyclovirobuxine D ameliorates cardiomyocyte senescence in diabetic cardiomyopathy mice by enhancing mitochondrial function via sirtuin 3–ATP5O signal axis
Source: Chin Med. 2025 Nov 13;20:187. doi: 10.1186/s13020-025-01254-3 (PMC12613623; doi:10.1186/s13020-025-01254-3)
Supplement: Supplementary file 1 — Additional file 1. [file 13020_2025_1254_MOESM1_ESM.docx]

**Supplementary Material**

**
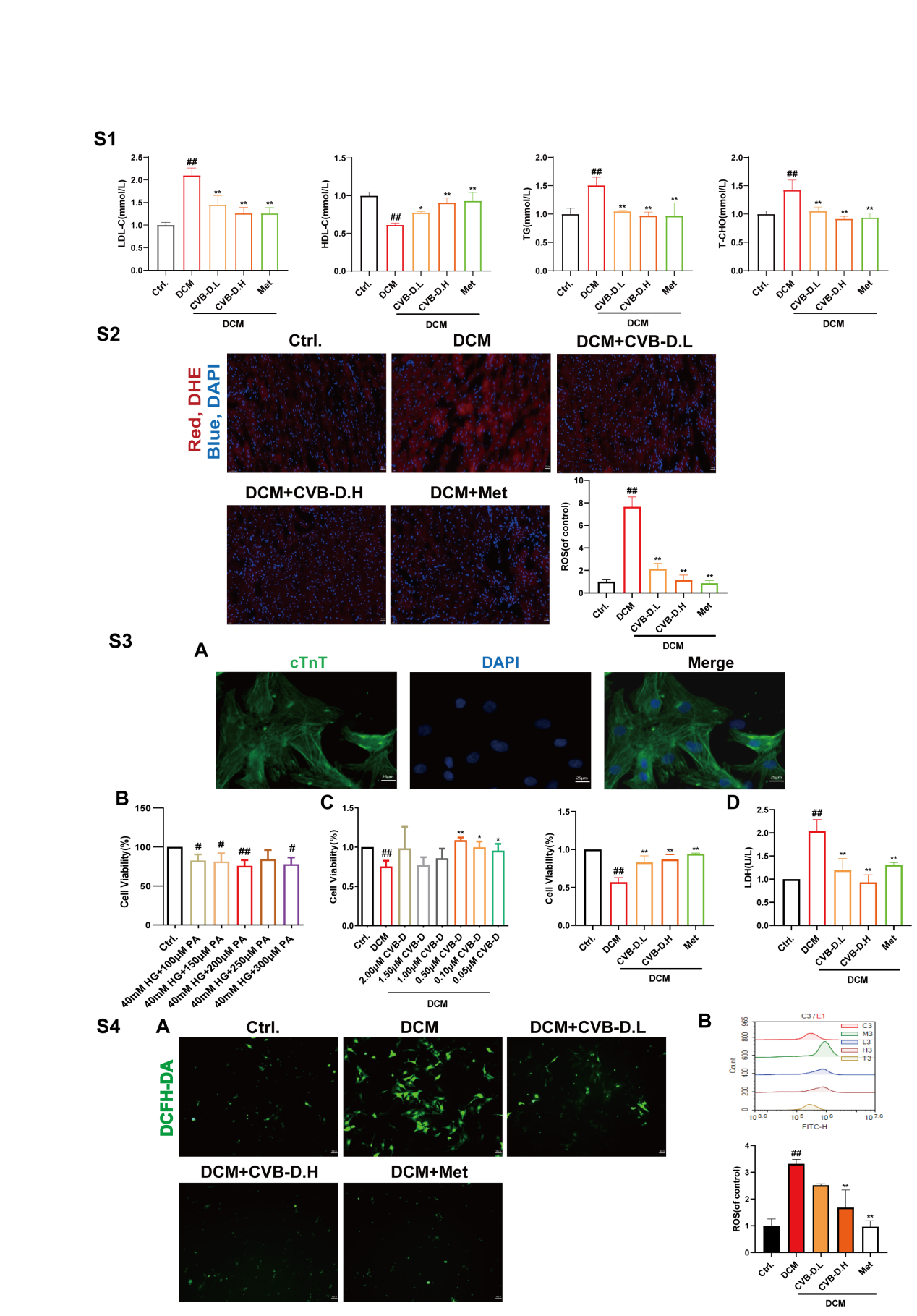
**

(S1) CVB-D on the serum levels of low-density lipoprotein (LDL), high-density lipoprotein (HDL), triglycerides (TG) and total cholesterol (TC) (n > 6). ^#^*p* < 0.05, ^##^*p* < 0.01 versus the control group; ^*^*p* < 0.05, ^**^*p* < 0.01 versus the model group.

(S2) Representative images of dihydroethidium (DHE) dyeing of the heart tissue from mice in each group (scale bar = 25 µm), and quantitative analysis of ROS levels (n = 6). ^#^*p* < 0.05, ^##^*p* < 0.01 versus the control group; ^*^*p* < 0.05, ^**^*p* < 0.01 versus the model group.

(S3 A) Representative images of immunofluorescence dyeing of CTnT in the NMVMs (scale bar = 25 µm). (S3 B) and (S3 C) Cell viability of each group was determined by MTT assay (n = 3). (S3 D) LDH activity (n = 3). ^#^*p* < 0.05, ^##^*p* < 0.01 versus the control group; ^*^*p* < 0.05, ^**^*p* < 0.01 versus the model group.

(S4 A) NMVMs were detected with a DCFH-DA fluorescent dyeing kit (scale bar = 50 µm). (S4 B) ROS of NMVMs was measured by flow cytometry, while the content of ROS was quantified (n = 3). ^#^*p* < 0.05, ^##^*p* < 0.01 versus the control group; ^*^*p* < 0.05, ^**^*p* < 0.01 versus the model group.

**
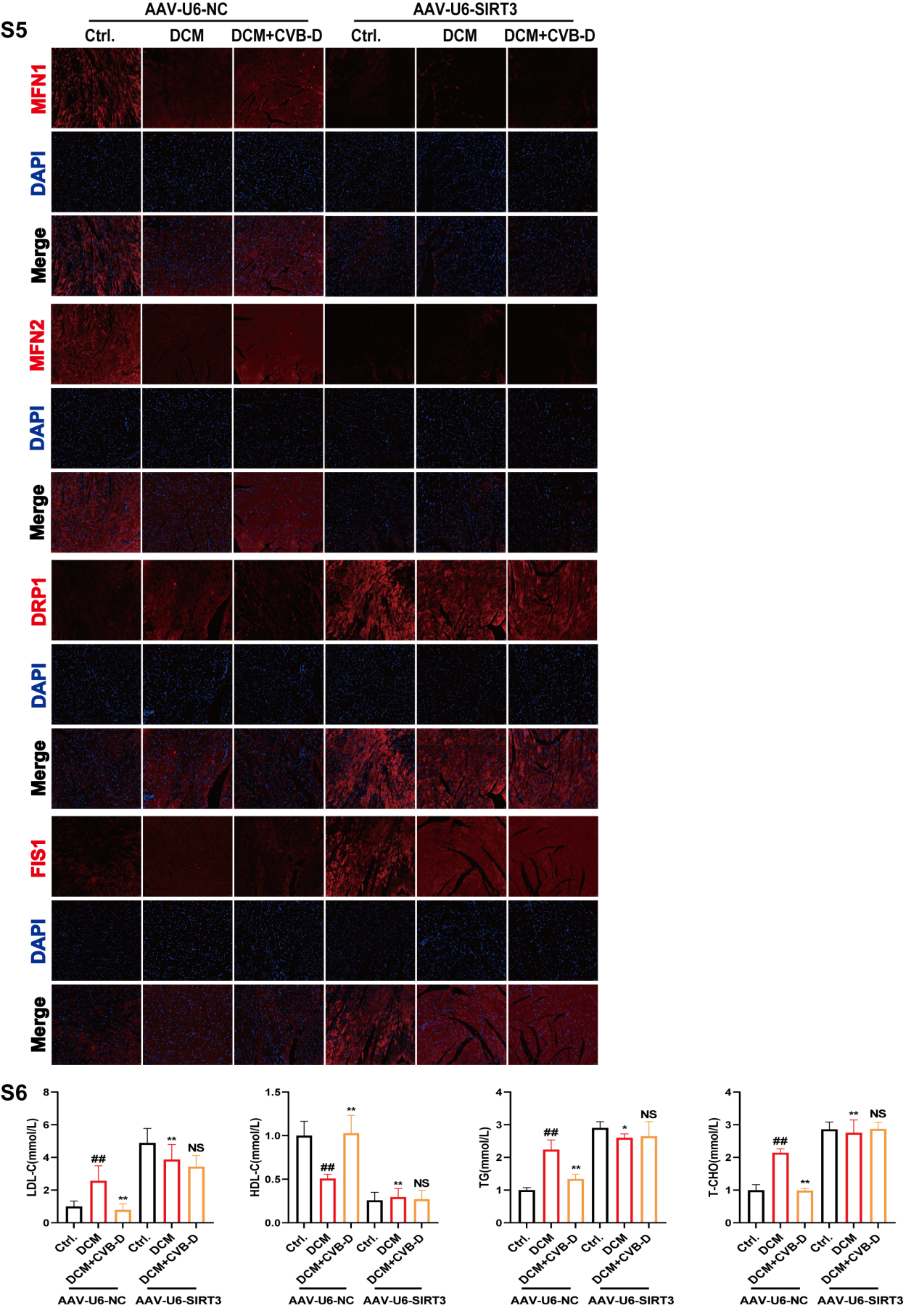
**

(S5) Immunofluorescence images of the mitochondrial dynamics-related proteins (scale bar = 20 µm).

(S6) and (S9) Detection of serum low-density lipoprotein (LDL), high-density lipoprotein (HDL), triglyceride (TG) and total cholesterol (TC) levels in mice (n = 6). ^#^*p* < 0.05, ^##^*p* < 0.01 versus the control group; ^*^*p* < 0.05, ^**^*p* < 0.01 versus the model group.

**
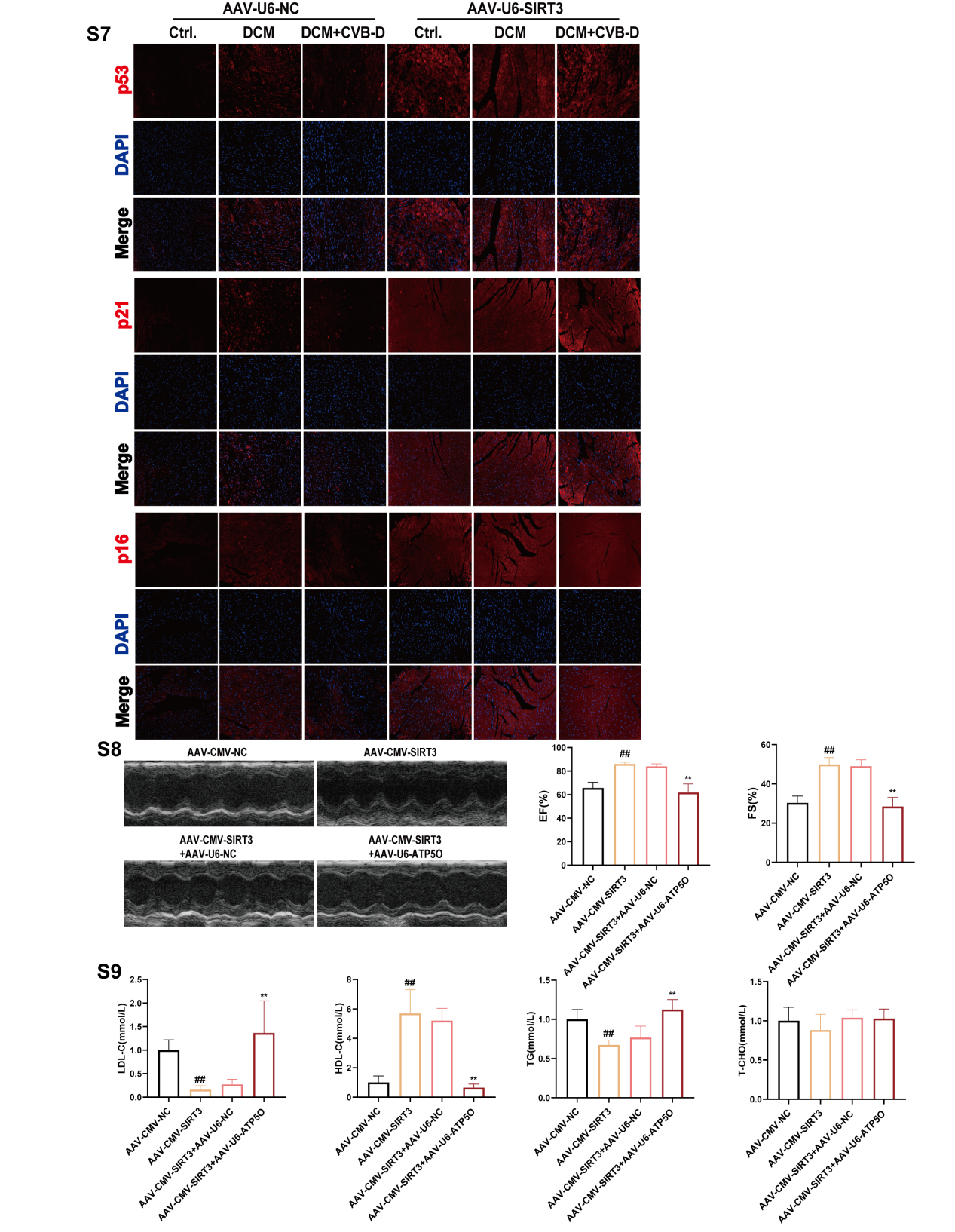
**

(S7) Immunofluorescence images of the p53, p21 and p16 proteins (scale bar = 20 µm).

(S8) Representative M-mode echocardiographic images and quantitative analysis of data for the ejection fraction (EF%) and fractional shortening (FS%) (n = 6). *^#^p* < 0.05, *^##^p* < 0.01 versus the AAV-CMV-NC group; *^*^p* < 0.05, *^**^p* < 0.01 versus the AAV-CMV-SIRT3 group.


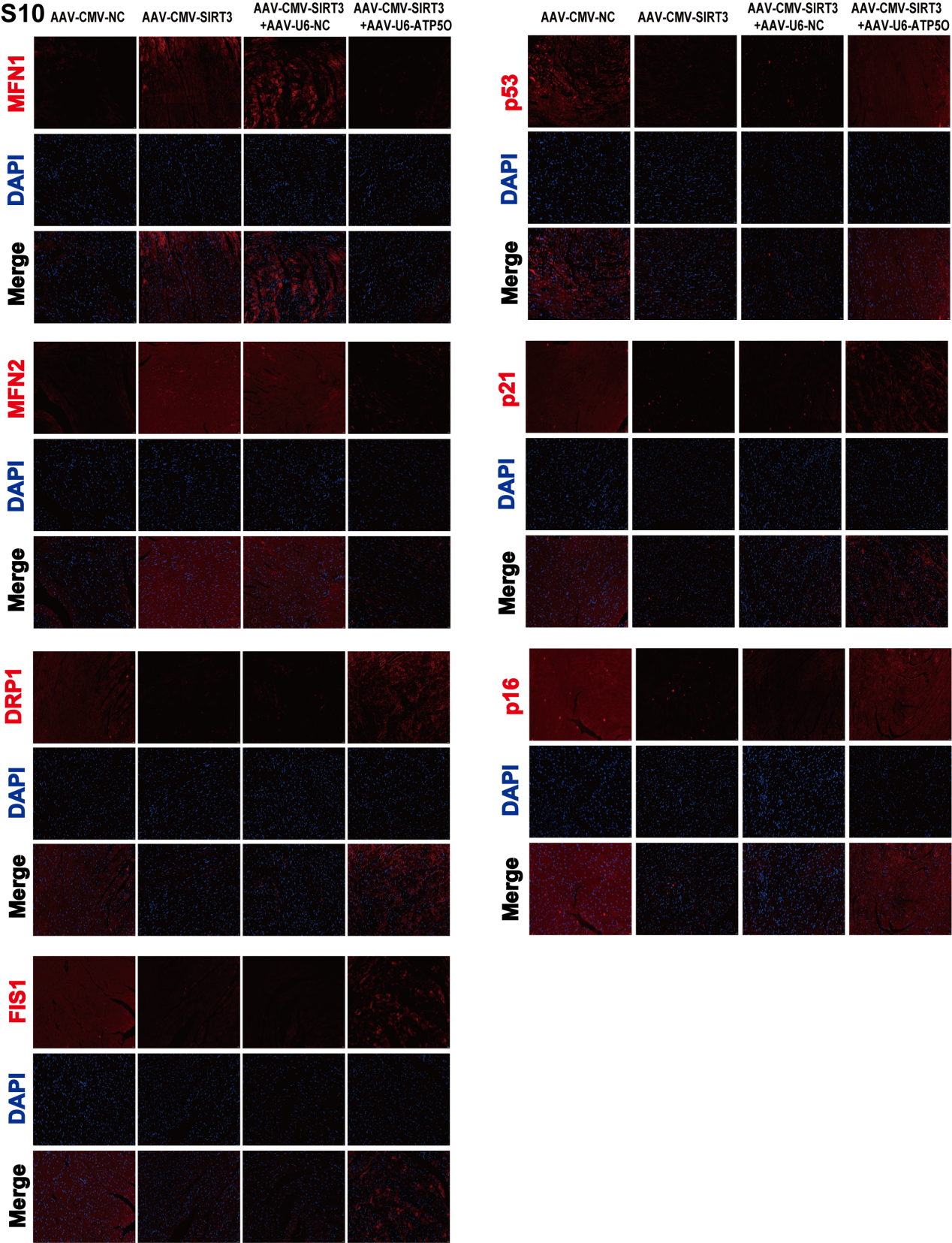


(S10) Immunofluorescence images of the mitochondrial dynamics-contacted and aging-contacted proteins (scale bar = 20 µm).
