# Supplementary material for: Cyclovirobuxine D ameliorates cardiomyocyte senescence in diabetic cardiomyopathy mice by enhancing mitochondrial function via sirtuin 3–ATP5O signal axis
Source: Chin Med. 2025 Nov 13;20:187. doi: 10.1186/s13020-025-01254-3 (PMC12613623; doi:10.1186/s13020-025-01254-3)
Supplement: Supplementary file 2 — Additional file 2. [file 13020_2025_1254_MOESM2_ESM.pdf]

Fig.2 G

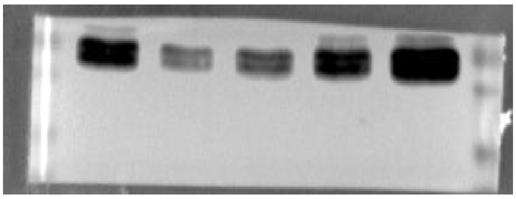

ATP5O

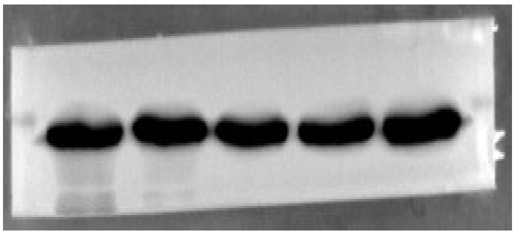

GAPDH

Fig.2 J

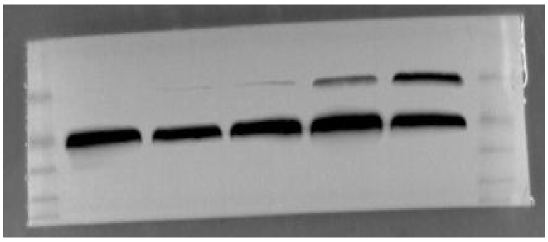

MFN1

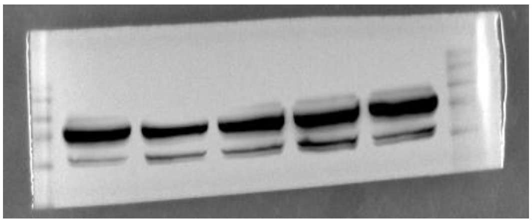

MFN2

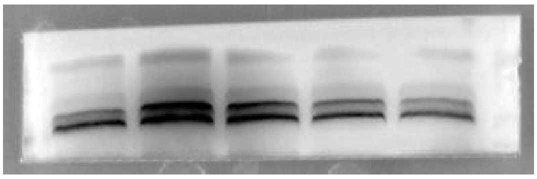

DRP1

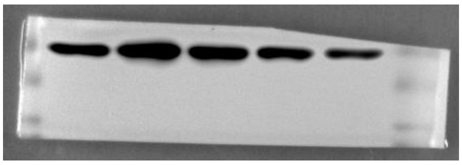

FIS1

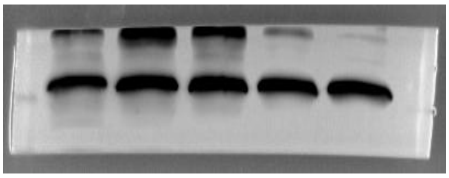

GAPDH

Fig.3 J

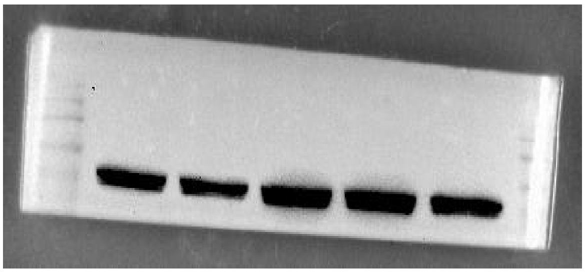

MFN1

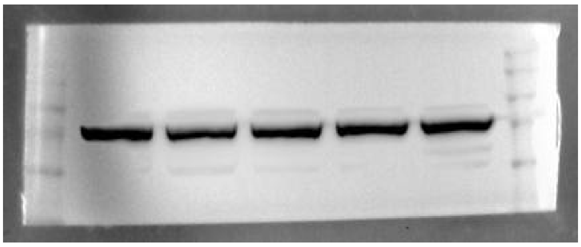

MFN2

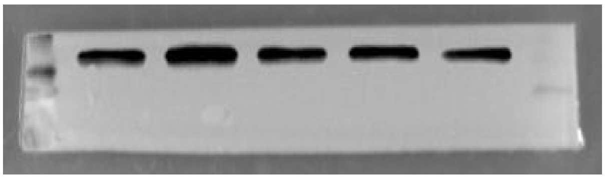

DRP1

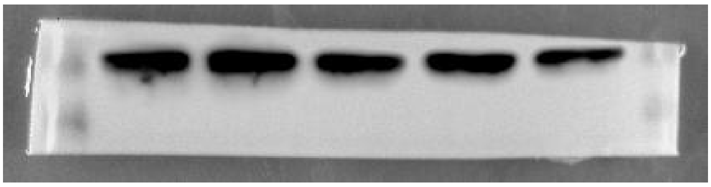

FIS1

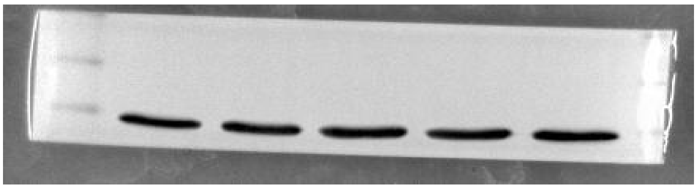

GAPDH

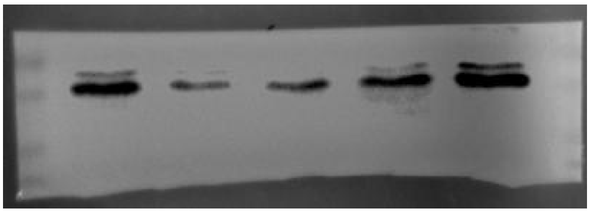

ATP5O

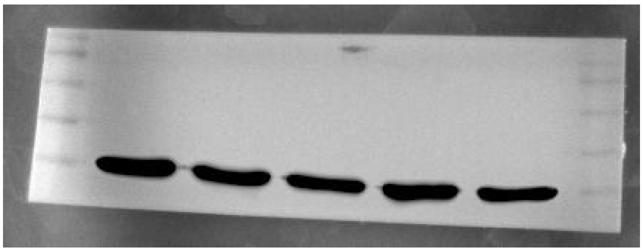

GAPDH

Fig.4 B

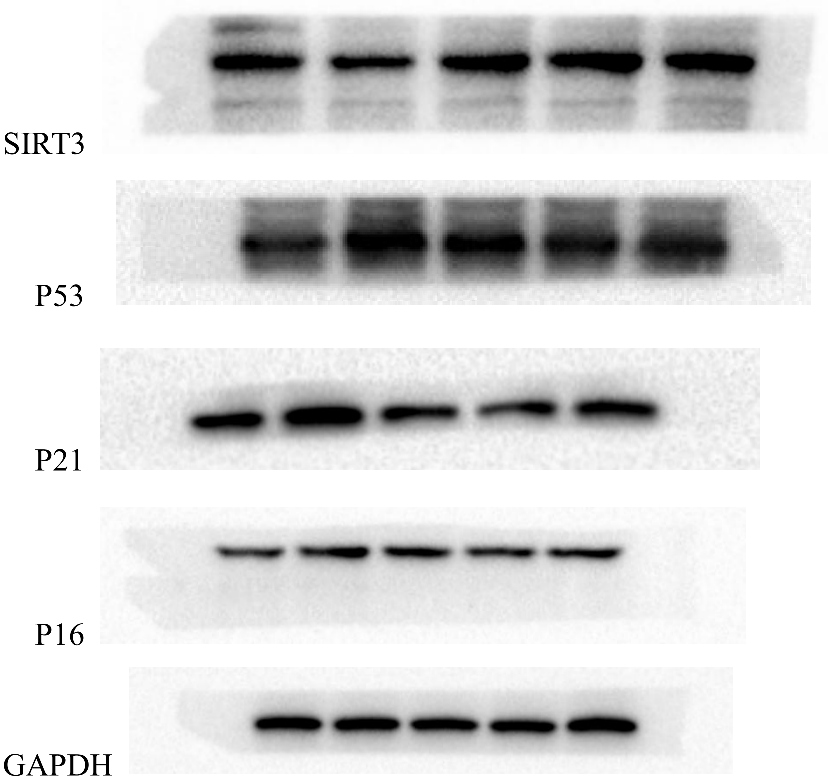

Fig.4 J

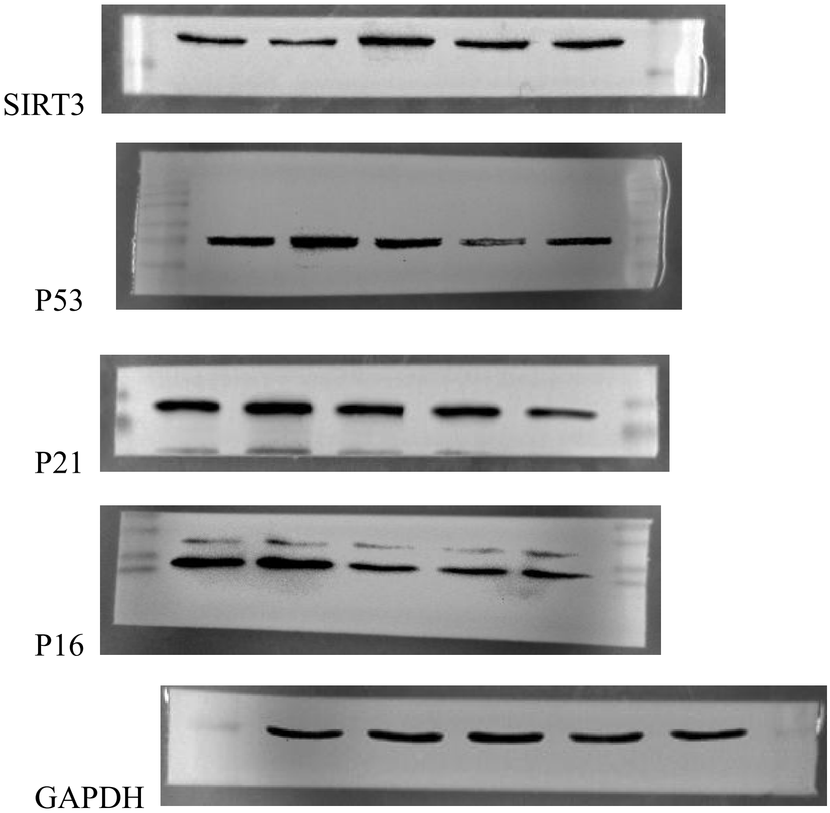

Fig.5 E

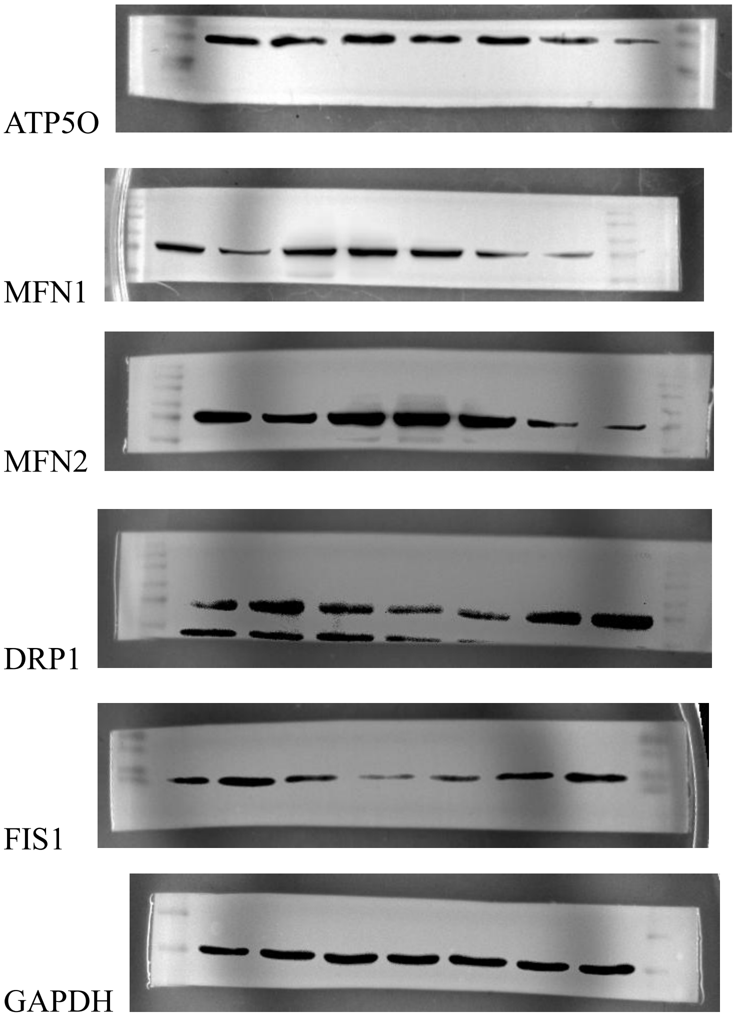

Fig.6 D

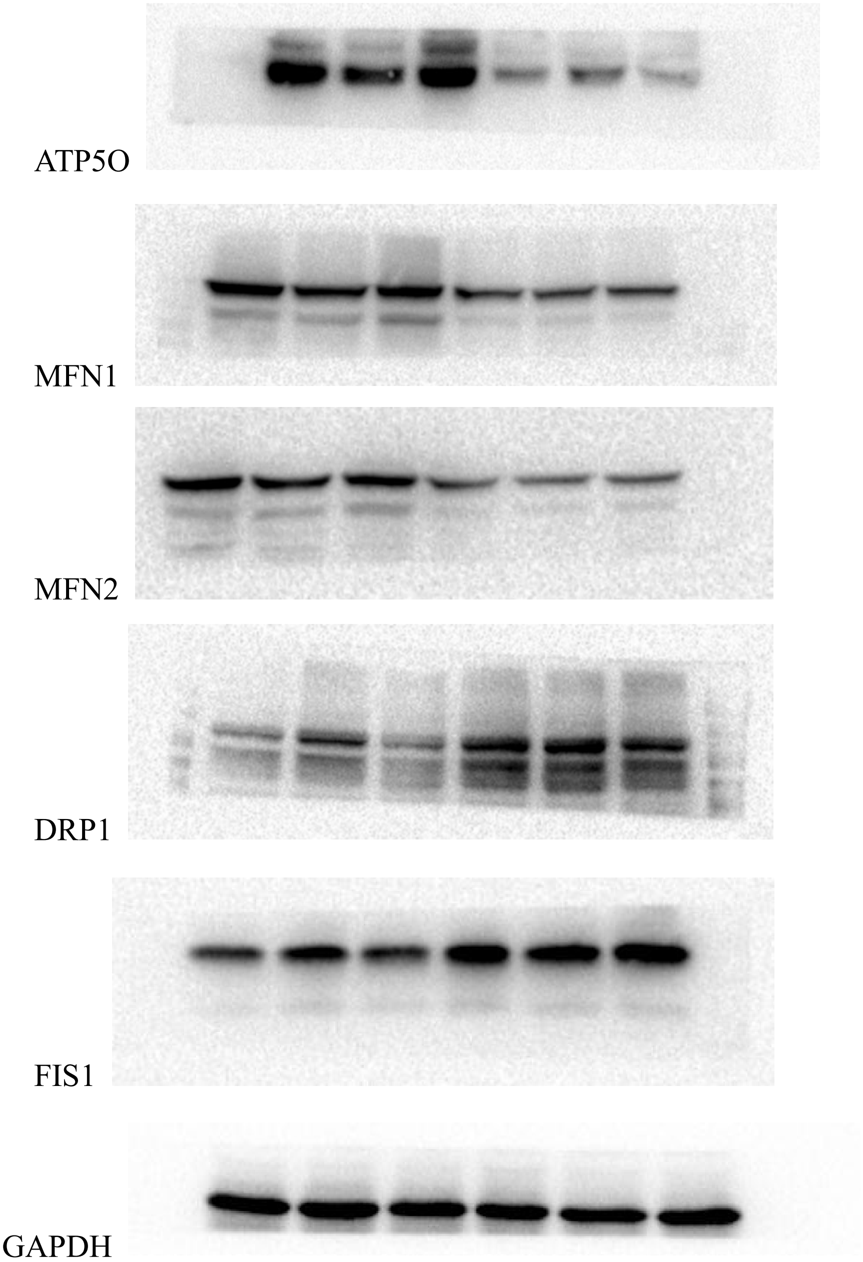

Fig.6 G

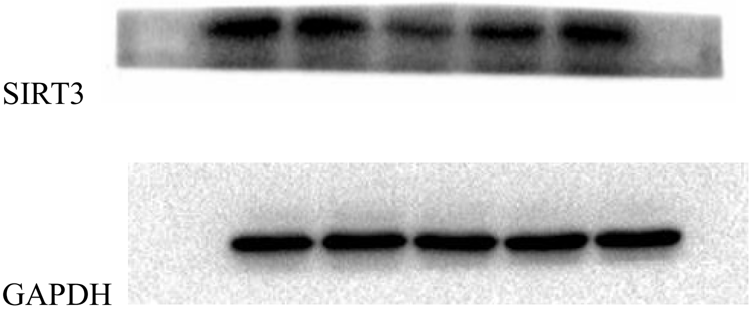

Fig.6 O

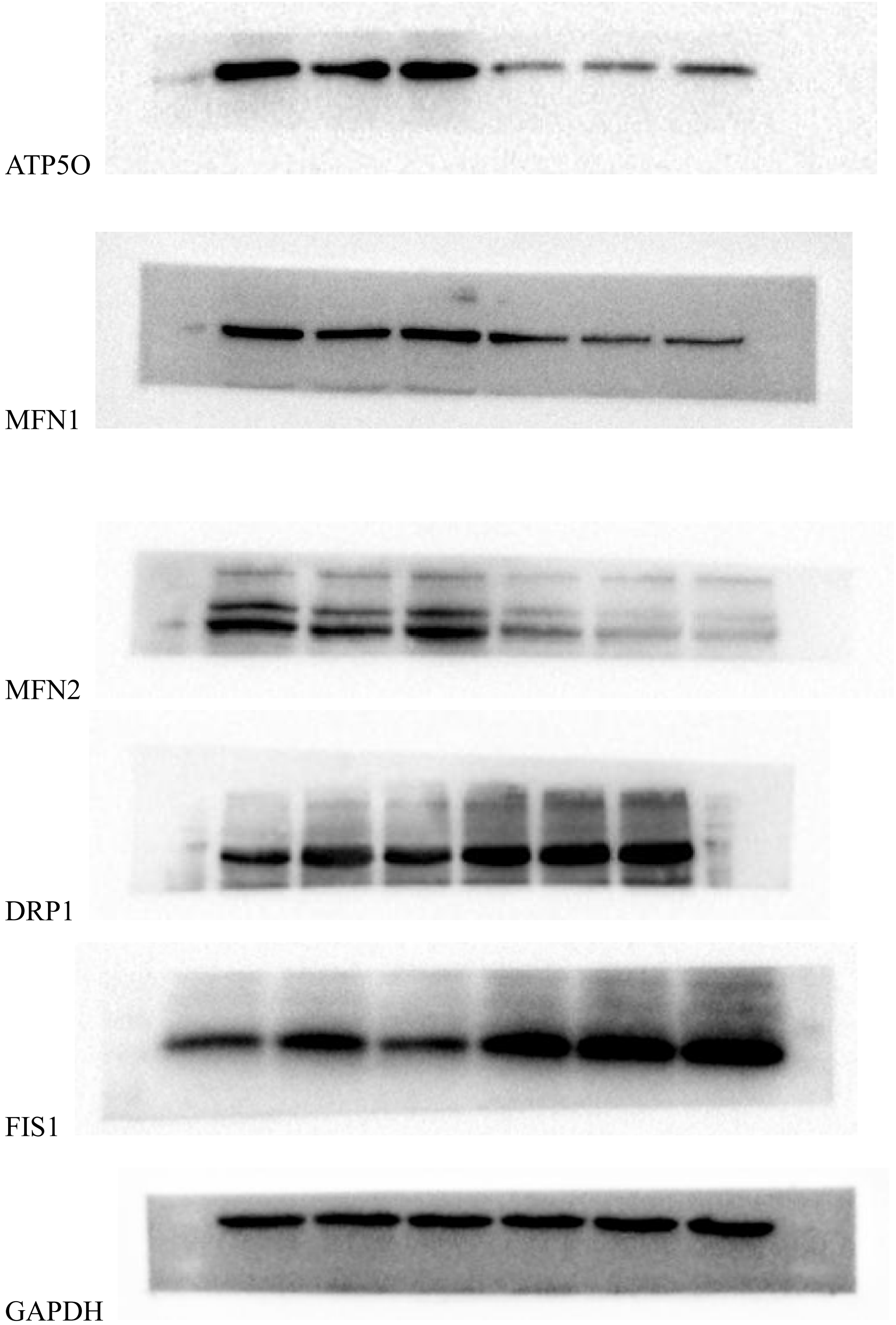

Fig.7 A

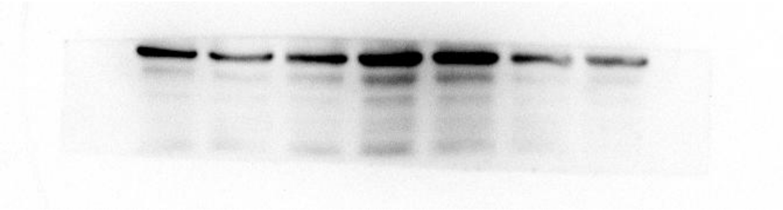

SIRT3

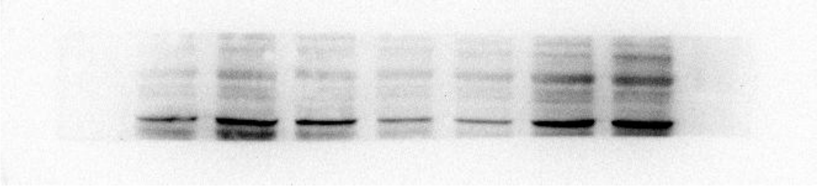

P53

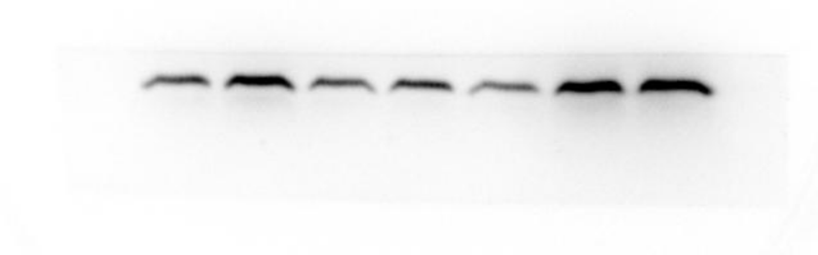

P21

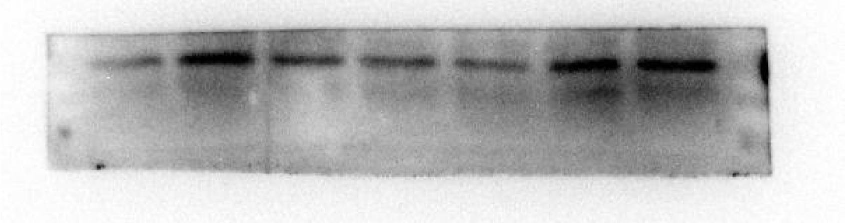

P16

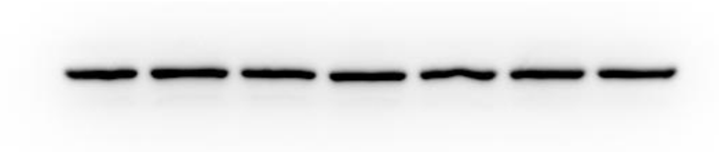

GAPDH

Fig.7 G

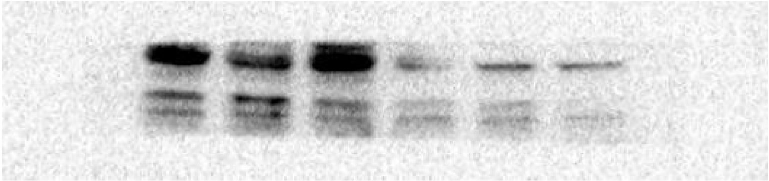

SIRT3

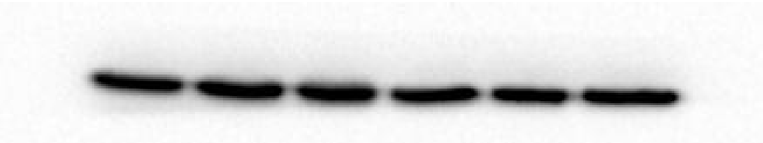

GAPDH

Fig.7 I

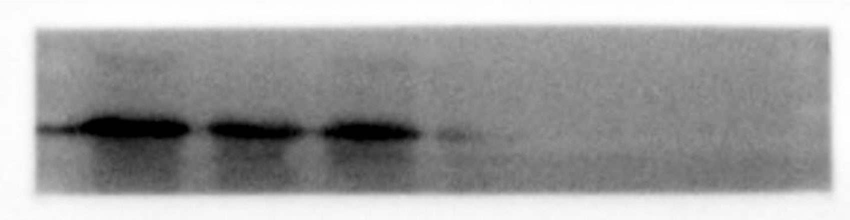

SIRT3

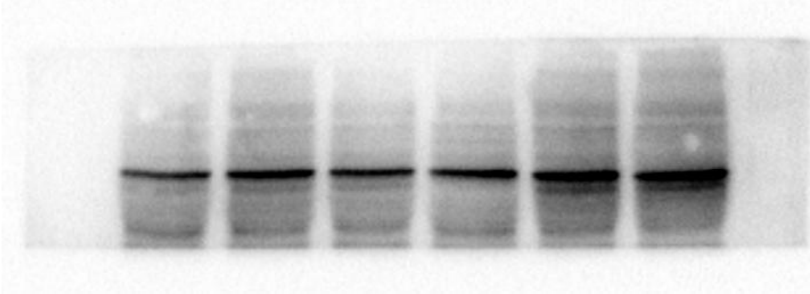

P53

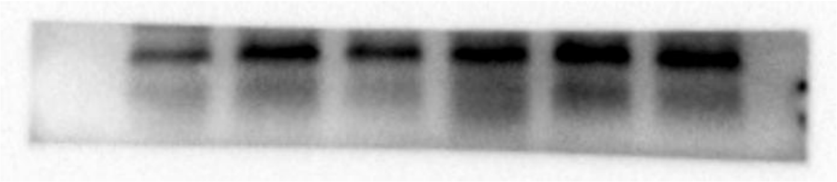

P21

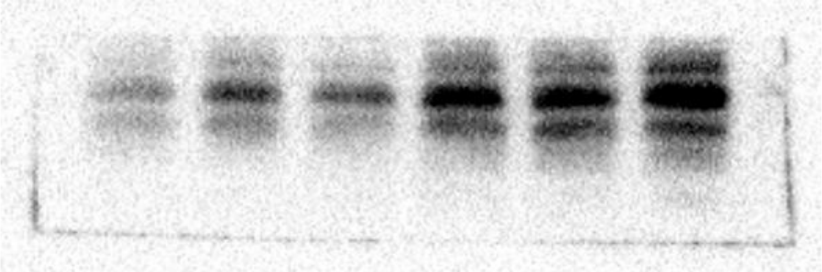

P16

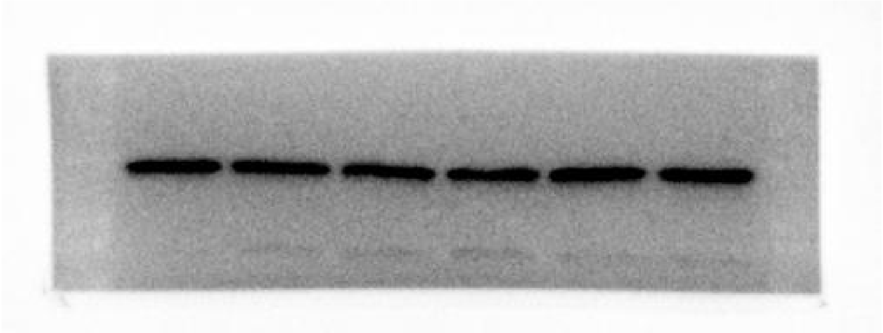

GAPDH

Fig.8 C

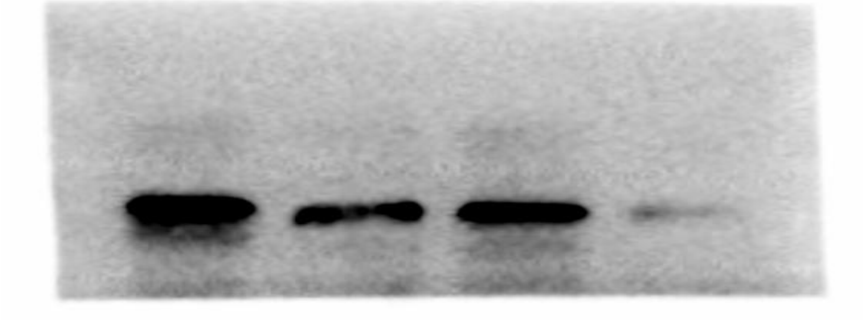

IB:SIRT3| SIRT3

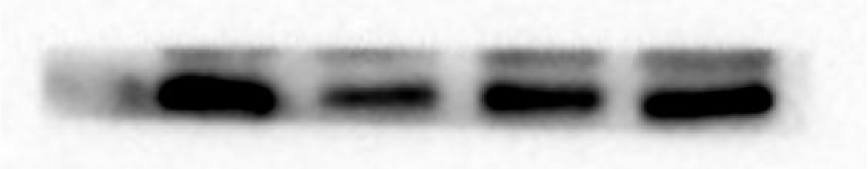

Lysate| SIRT3

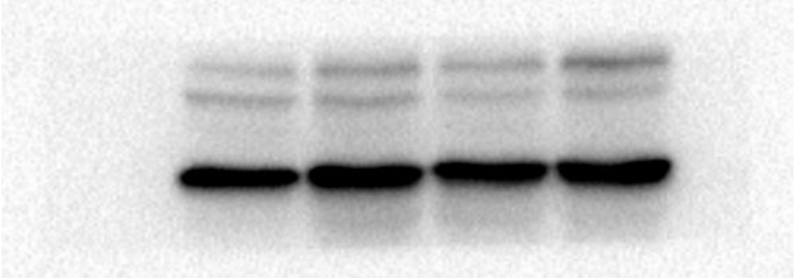

Lysate| GAPDH

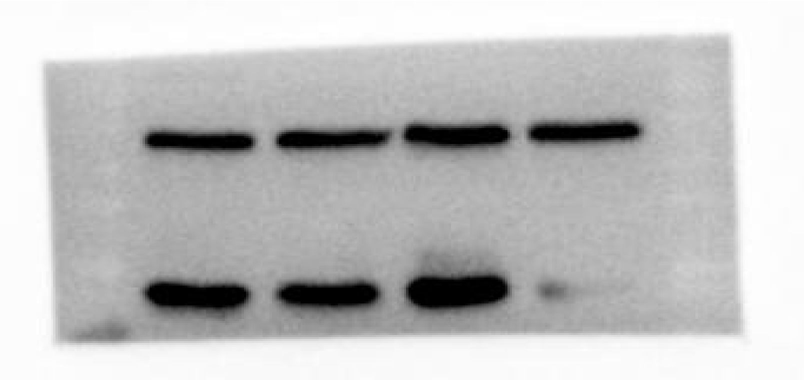

IB:ATP5O| ATP5O

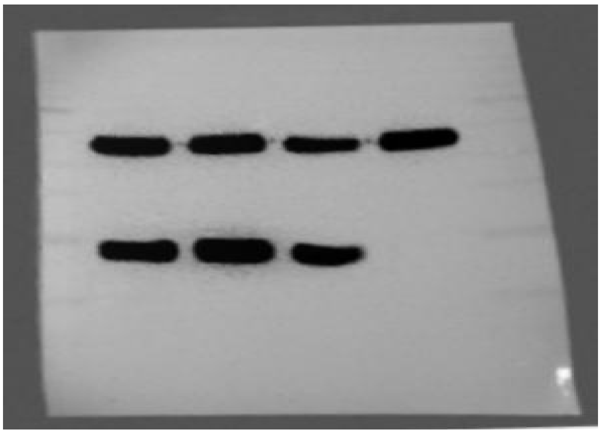

IB:ATP5O| ATP5O

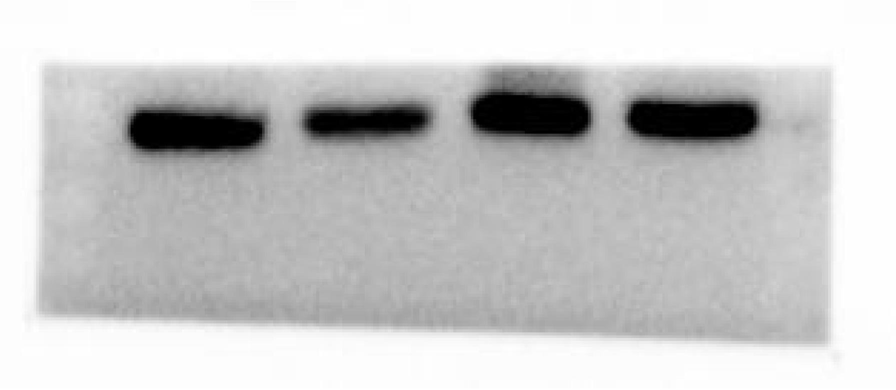

Lysate| ATP5O

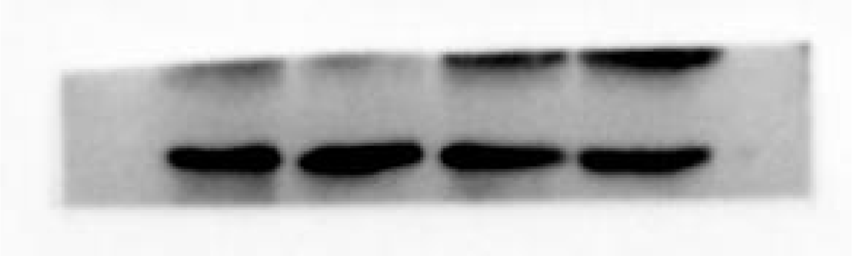

Lysate| GAPDH

Fig.8 F

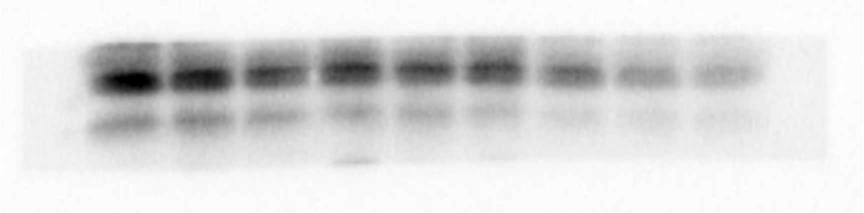

CVB-D| SIRT3

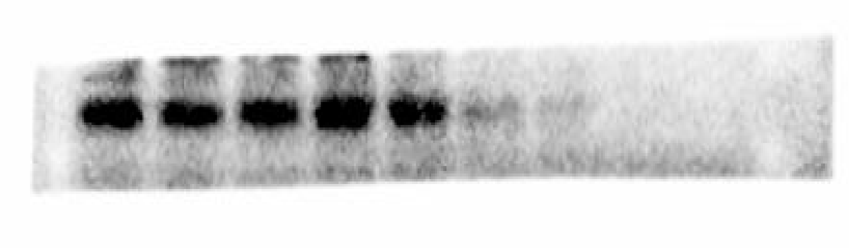

DMSO| SIRT3

Fig.9 B

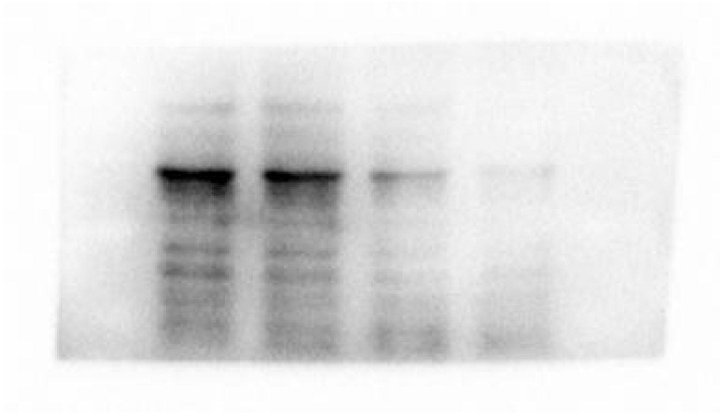

IB:ATP5O

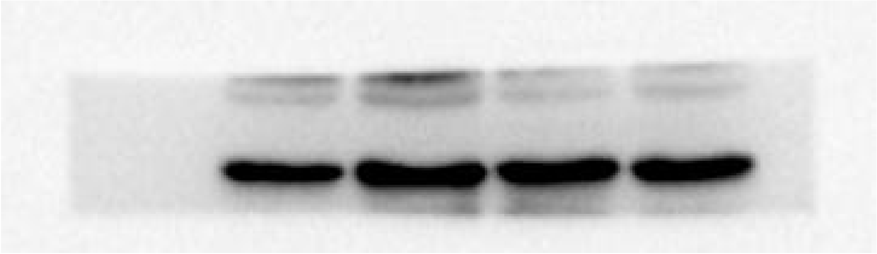

Input

Fig.9 C

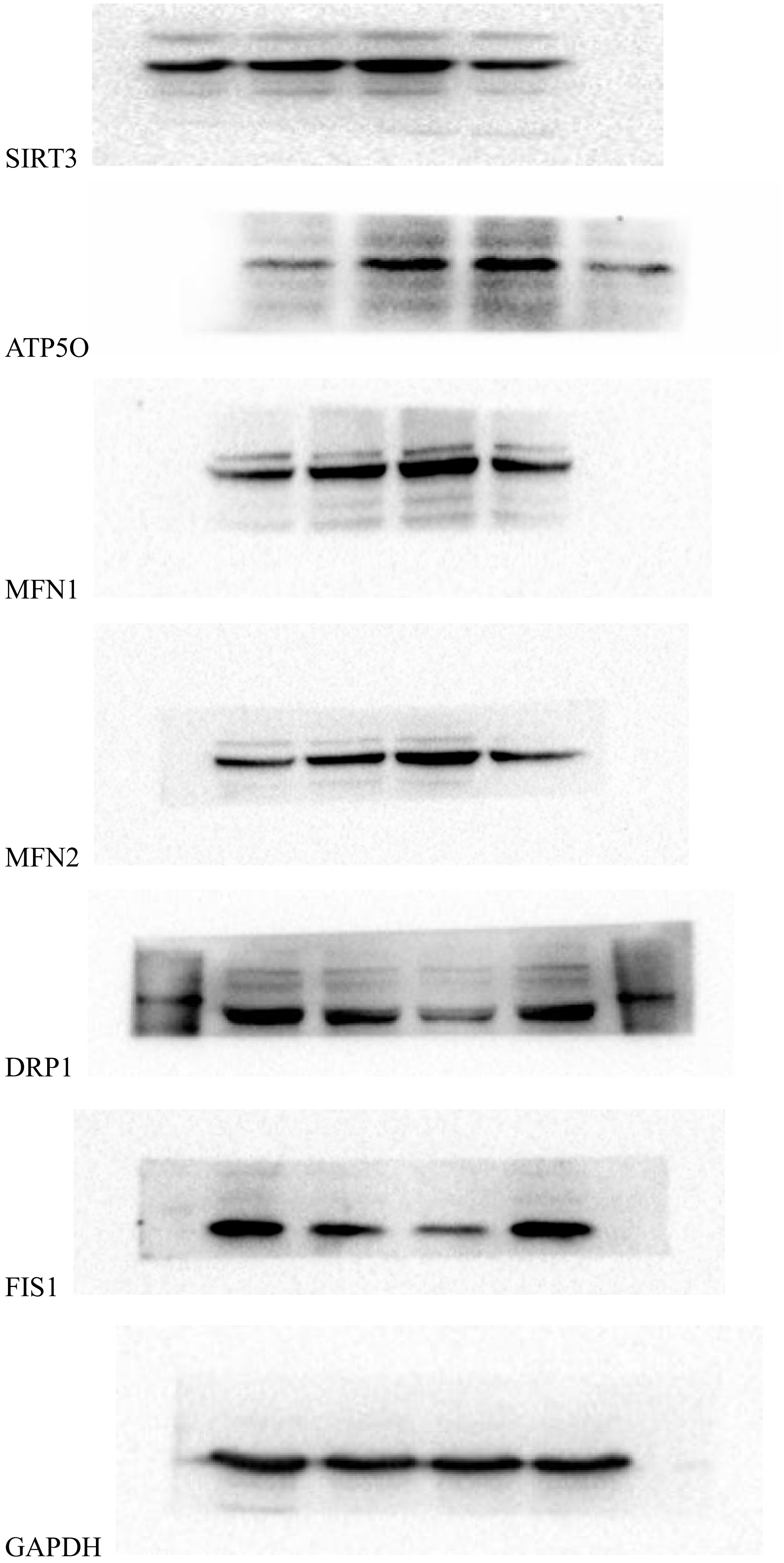

Fig.10 A

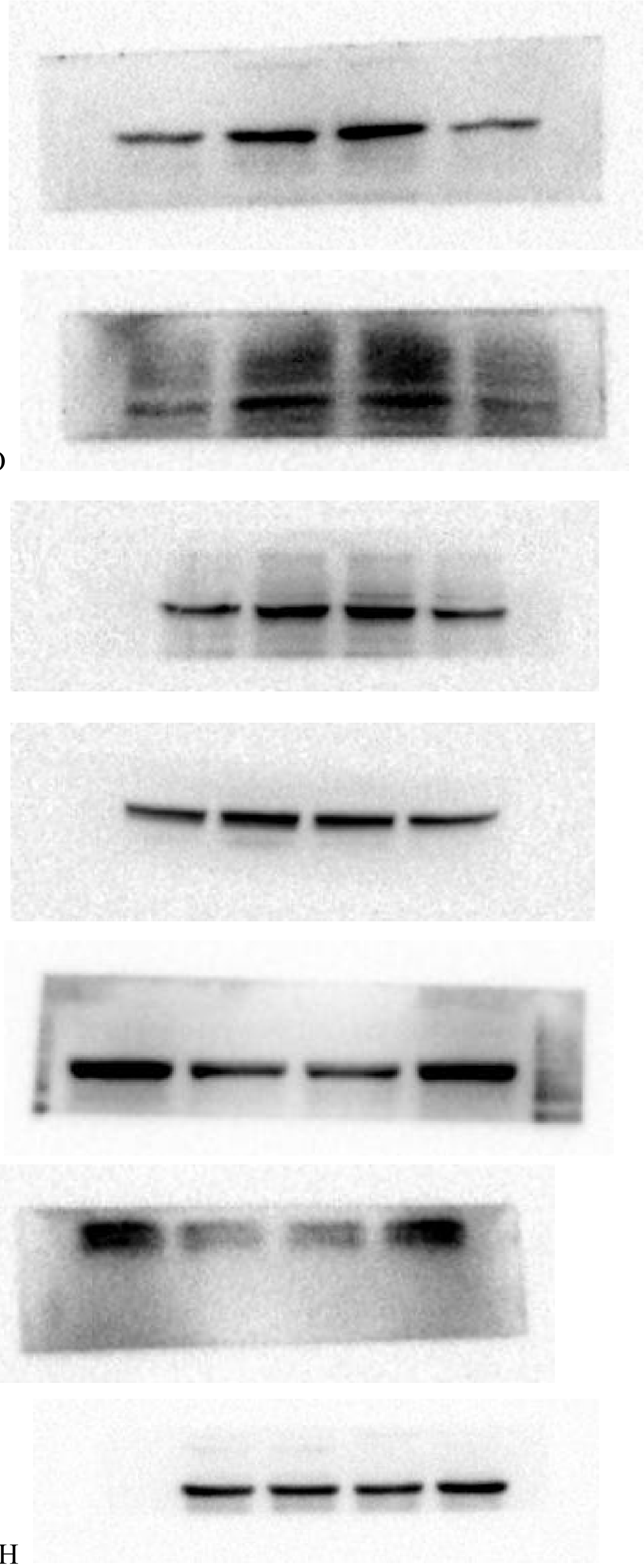

SIRT3

ATP5O

MFN1

MFN2

DRP1

FIS1

GAPDH

Fig.10 I

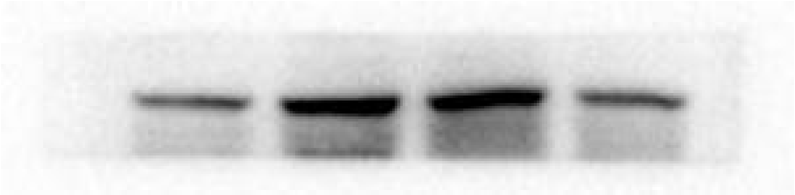

SIRT3

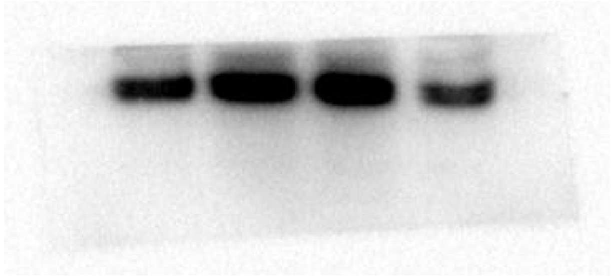

ATP5O

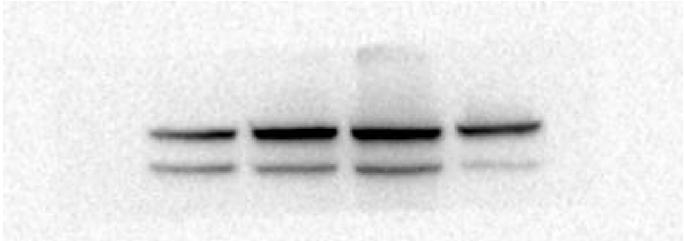

MFN1

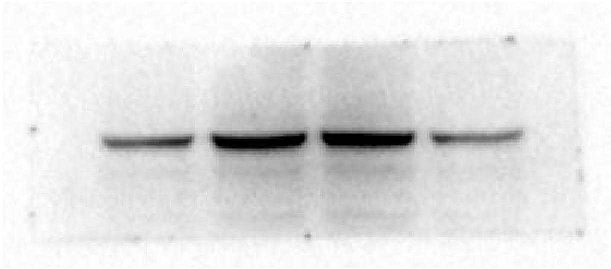

MFN2

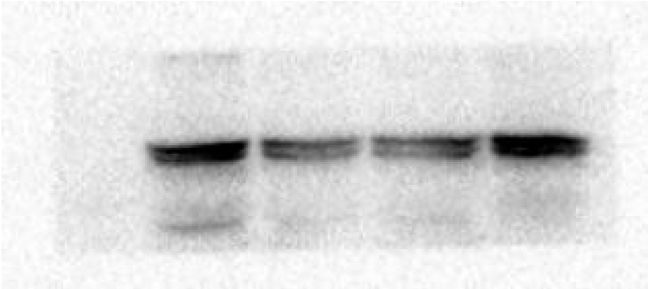

DRP1

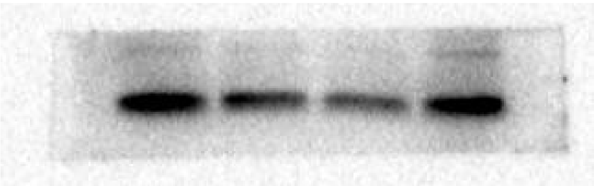

FIS1

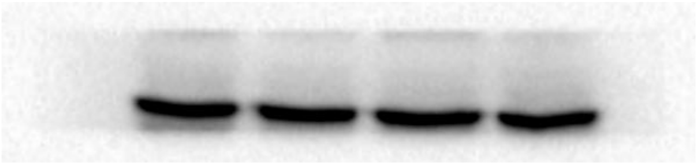

GAPDH
