# Supplementary material for: Cyclovirobuxine D ameliorates cardiomyocyte senescence in diabetic cardiomyopathy mice by enhancing mitochondrial function via sirtuin 3–ATP5O signal axis
Source: Chin Med. 2025 Nov 13;20:187. doi: 10.1186/s13020-025-01254-3 (PMC12613623; doi:10.1186/s13020-025-01254-3)
Supplement: Supplementary file 3 — Additional file 3. [file 13020_2025_1254_MOESM3_ESM.docx]

**Supplementary Material 3**


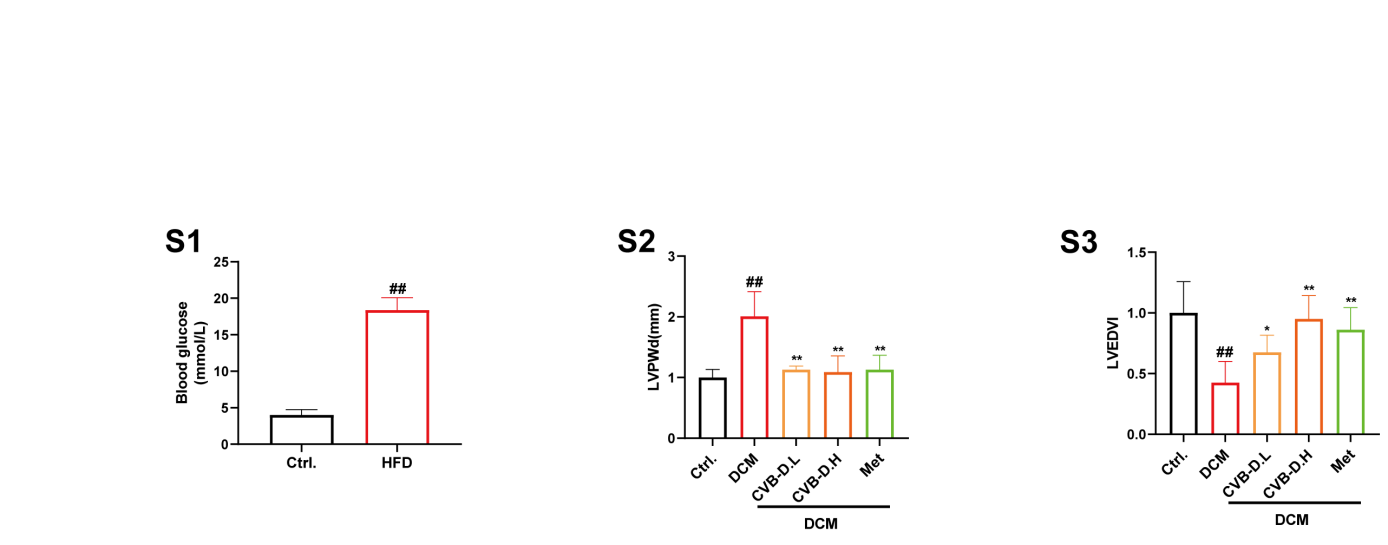


(S1) Blood glucose of the mice (n = 6). (S2-S3) Representative M-mode echocardiographic images and data analysis of the LVPWd and LVEDVI (n = 6).
